# The delirium dichotomy of remimazolam: a differential risk profile for emergence delirium versus postoperative delirium in surgical patients: a systematic review and meta-analysis

**DOI:** 10.3389/fmed.2026.1841225

**Published:** 2026-05-11

**Authors:** Ziyu Zhu, Sizu Wang, Xizhi Gu, Jianping Kong, Ying Zhang, Liqun Yang, Xibing Ding, Weifeng Yu

**Affiliations:** 1 Department of Anaesthesiology, Renji Hospital, School of Medicine, Shanghai Jiaotong University, Shanghai, China; 2 Xuzhou Medical University, Xuzhou, Jiangsu, China; 3 Department of Anaesthesiology, Dayao People’s Hospital, Chuxiong, Yunnan, China

**Keywords:** benzodiazepine, emergence delirium, meta-analysis, postoperative delirium, remimazolam, systematic review

## Abstract

**Background:**

The association between remimazolam, a novel ultra-short-acting benzodiazepine, and the risk of postoperative delirium (POD) and emergence delirium (ED) remains controversial, particularly following prolonged infusion.

**Methods:**

PUBMED, EMBASE, WEB OF SCIENCE and the Cochrane Library electronic databases were searched up to December 10, 2025. The primary outcome was the incidence of delirium. Secondary outcomes included postoperative nausea and vomiting (PONV), respiratory depression after extubation, extubation time and length of hospital stay. Subgroup and meta-regression analyses were conducted to assess clinical and methodological sources of heterogeneity in intervention effect, including age, type of surgery, assessment methods of delirium, depth of anesthesia monitoring, the use of flumazenil as an antagonist for remimazolam.

**Results:**

A total of 30 trials were included, consisting of 25 RCTs, 4 retrospective studies and a prospective cohort study. The incidence of delirium was 11.4% (312/2734) in the remimazolam group and 15.2% (429/2827) in the non-remimazolam group, showing no significant difference (RR = 0.81; 95% confidence interval (CI), 0.63–1.05, *p* = 0.11) between groups. Subgroup analysis by anesthesia type, however, revealed a significant effect modification. In patients undergoing general anesthesia, remimazolam was associated with a 23% reduction in the risk of delirium (RR = 0.77, 95% CI: 0.60–1.00, *p* = 0.05). When viewed in terms of this dichotomy, no significant difference was observed in 22 studies evaluating the incidence of POD between remimazolam group (13.1%, 295/2260) and non-remimazolam group (16%, 390/2431) (RR = 0.93; 95% CI, 0.76–1.15, *p* = 0.52), either in 8 studies on incidence of emergence delirium (RR = 0.43; 95% CI, 0.13–1.37, *p* = 0.15). The pooled analysis using a fixed-effect model showed that remimazolam was associated with a statistically significant increase in the risk of PONV compared to non-Remimazolam groups (RR = 1.20, 95% CI: 1.02–1.42; *p* = 0.03). Other secondary outcomes, respiratory depression after extubation (RR = 0.96; 95% CI, 0.63–1.44, *p* = 0.84), extubation time (MD = -1.30, 95% CI: −3.46-0.85, *p* = 0.24) and length of hospital stay (MD = 0.08, 95% CI: −0.28-0.44, *p* = 0.65) showed no significant difference between remimazolam group and non-remimazolam group.

**Conclusion:**

In this systematic review and meta-analysis, prolonged continuous intravenous administration of remimazolam throughout the surgical procedure does not increase the risk of delirium compared to other anesthetic regimens. In addition, remimazolam has potential benefits in the pediatric population as it reduces the risk of ED.

**Systematic review registration:**

https://www.crd.york.ac.uk/PROSPERO/view/CRD420251138775, Identifier: CRD420251138775.

## Introduction

Postoperative delirium (POD) and emergence delirium (ED) represent significant clinical challenges in anesthesiology and perioperative practice. They are characterized by fluctuating disturbances in attention, consciousness, and cognition. While POD and ED both representing acute perioperative cognitive dysfunction, differ primarily in their temporal profiles. This study explores a potential dichotomy in remimazolam’s effects: its differential risk profile for ED, an immediate post-anesthesia phenomenon occurring in the post-anesthesia care unit (PACU), versus POD, which manifests days following surgery. Considering that postoperative delirium and emergence delirium may share certain core pathophysiological mechanisms, such as the neuroinflammation theory, ED could represent the acute manifestation of neuroinflammation, whereas POD may be a consequence of persistent or chronic inflammatory responses. We tend to regard perioperative brain health as an integrated management goal and establish a continuous delirium monitoring process from the PACU to the ward.

Delirium not only prolongs hospital stays and increases healthcare costs but also is strongly associated with long-term cognitive decline and elevated mortality (1–3), with the high incidence of POD as 15–50% (4–6) and ED as 10%–20% (7–9). With the continuous rise in global surgical volumes and an aging population, identifying effective strategies to prevent and manage postoperative delirium has become a priority in anesthesiology and perioperative research.

In recent years, the role of benzodiazepines in delirium prevention has garnered significant attention. Traditional benzodiazepines were believed to increase the risk of delirium (10). However, the emergence of remimazolam, a novel ultra-short-acting benzodiazepine, has challenged this notion. Remimazolam is a water-soluble, rapidly acting sedative that functions as a positive allosteric modulator of the *γ*-aminobutyric acid type A (GABA_A_) receptor (11, 12). Its unique pharmacological properties include rapid onset (about 1–2 min), short duration with half-life of 37 min, metabolism independent of hepatic or renal function, inactive metabolites, and reversibility with flumazenil (13, 14). Compared to conventional benzodiazepines, remimazolam offers more predictable recovery times and superior hemodynamic stability, maintaining better cerebral oxygen supply–demand balance. Despite some encouraging findings suggesting its advantages on delirium, critical questions remain regarding its optimal dosing, timing, and administration strategies, its efficacy across different surgical types and patient populations, and potential synergistic effects with other anesthetics.

This systematic review and meta-analysis aim to clarify whether remimazolam is associated with a lower incidence of delirium compared to other commonly used sedatives. Our findings may help the development of safer and more effective sedation strategies for patients undergoing surgery, thereby improving postoperative outcomes and reducing the burden of POD and ED.

## Methods

The conduct and reporting of this systematic review and meta-analysis adhered to the PRISMA 2020 guidelines (15). Prior to data extraction, the study protocol was prospectively registered with the International Prospective Register of Systematic Reviews (PROSPERO; Registration ID: CRD420251138775), which details the predefined methodology.

### Date sources and search strategy

PUBMED, EMBASE, WEB OF SCIENCE and the Cochrane Library electronic databases were searched from inception to January 5, 2025. The search was updated on December 10, 2025. A combination of the following terms were used: (1) “remimazolam” OR “ONO 2745” OR “ONO2745” OR “CNS7056” OR “BYFAVO”; (2) “propofol” OR “dexmedetomidine” OR “sevoflurane” OR “isoflurane” OR “desflurane” OR “Hypnotics and Sedatives”; (3) “Anesthesia, General” OR “anesthesia” OR “anaesthesia” OR “Anesthesia and Analgesia”; (4) “Delirium” OR “delirium” OR “Postoperative Delirium” OR “Emergence Delirium” OR “Postoperative Cognitive Dysfunction” OR “POD” OR “ED”; and (5) “randomized controlled trial” OR “controlled clinical trial” OR “clinical trials” OR “trial.” The detailed search strategy for each database is presented in Supplementary data. In addition to the electronic searches, manual identification methods were employed to ensure comprehensive coverage. These included a systematic examination of reference lists from the included articles and relevant previous reviews.

### Inclusion criteria

Eligible studies were selected based on the following PICOS (participants, interventions, comparators, outcomes, and study design) criteria:

(1) *Population*: Restricted to hospitalized patients, requiring surgery.
(2) *Intervention*: Use of remimazolam as the primary hypnotic or as an adjunct, for the continuous maintenance of anesthesia throughout the duration of the surgical procedure.
(3) *Comparison*: Other sedative hypnotics, such as propofol, inhalation anesthetics, dexmedetomidine, and saline.
(4) *Outcome*: At least one of the primary outcomes, including the incidence of postoperative delirium or emergency delirium. Secondary outcomes included postoperative nausea and vomiting, respiratory depression after extubation, extubation time and length of hospital stay.
(5) *Study design*: Randomized controlled trial, prospective study, or retrospective study;
(6) Published full-text, peer-reviewed clinical trials were included in the meta-analysis. All the included studies were published in English. Patients with no age limit having all types of anesthesia were included if the trial had delirium as an outcome as assessed by a validated method.

### Exclusion criteria

The studies were excluded based on the following criteria: (1) routine painless gastrointestinal endoscopy, bronchoscopy examinations, and other similar minor procedures; (2) use of remimazolam for the postoperative treatment of delirium or agitation; (3) studies were excluded if the reported primary outcome was described solely as ‘emergence agitation’ without a clear link to a validated delirium assessment tool or diagnostic framework; (4) the incidence of delirium, as a primary outcome, has not been reported; (5) remimazolam for sedation in mechanically ventilated ICU patients.

### Study selection and data extraction

The literature search and study selection process was executed by two investigators. The initial phase involved a duplicate check and screening of titles and abstracts to exclude clearly irrelevant records. Subsequently, the same two investigators independently performed a full-text review of the remaining articles for eligibility assessment. Any discrepancies arising during this stage were resolved through consensus discussion with a senior third investigator. Following study inclusion, two team members independently extracted summary data using a standardized form. The extracted variables encompassed: first author, publication year, country, study design, sample size, participant demographics, surgical type, ASA physical status, details of the anesthetic protocols (induction and maintenance doses for remimazolam and propofol), duration of follow-up, and the specific instrument used for delirium diagnosis.

The primary outcome was the incidence of postoperative delirium. This outcome was operationalized using any validated assessment method with documented sensitivity and specificity exceeding 80% relative to a reference standard diagnosis made using Diagnostic and Statistical Manual of Mental Disorders (DSM) or Classification of Diseases (ICD) criteria. Secondary outcomes included postoperative nausea and vomiting (PONV), Respiratory depression after extubation, extubation time and length of hospital stay.

### Risk of bias assessment

The methodological quality of the included studies was independently assessed by two reviewers using the revised Cochrane risk-of-bias tool (RoB 2) (16). This assessment addressed six key domains: random sequence generation, allocation concealment, blinding of participants and personnel, blinding of outcome assessment, incomplete outcome data, and selective reporting. For each domain, the risk of bias was judged as “low,” “unclear,” or “high.” Any discrepancies between the reviewers’ judgments were resolved through consensus discussion or, when necessary, arbitration by a third reviewer.

### Statistical analysis

For all meta-analyses, we generated forest plots to display the effect estimates alongside their 95% confidence intervals (CI). For continuous outcomes, the mean difference (MD) served as the summary measure. Heterogeneity among study results was quantified using the *I*^2^ statistic and assessed for significance using Cochran’s *Q* test. In this meta-analysis, model selection hinges on the extent of between study heterogeneity. Use a fixed-effect model when assuming all studies share a single true effect size; this is appropriate when clinical and methodological characteristics are highly consistent and statistical tests (e.g., *I*^2^ < 50%, *Q*-test *p* > 0.1) indicate negligible heterogeneity. Conversely, adopt a random-effects model when heterogeneity is present or suspected-evidenced by *I*^2^ > 50%, *Q*-test *p* < 0.1, or substantive clinical differences among studies, and wish to generalized the pooled estimate to a broader population of studies, acknowledging that the true effect size varies across studies. For studies reporting continuous outcomes as medians with interquartile ranges, these data were converted to means and standard deviations using established methods (17) to permit pooling in the meta-analysis. This approach is widely accepted for integrating such data when the distribution is approximately normal and the sample size is sufficient.

Meta-regression was performed to explore the sources of heterogeneity when significant heterogeneity was present in the primary analysis. Meta-regression assessed the impact of study-level covaries. Model fit was assessed with the adjusted R-squared (higher values reflect better fit). We assessed multicollinearity among the covariates using variance inflation factors (VIF). A VIF greater than 5 was considered indicative of problematic multicollinearity, and such variables were excluded from the final model. Subgroup analyses were conducted to identify potential sources of heterogeneity. Variables like country, types of delirium, surgery type, depth of anesthesia monitoring, assessment methods of delirium, age and the use of flumazenil as an antagonist were examined. A leave-one-out sensitivity analysis was performed to assess small-study effects and determine whether any individual study significantly influenced the robustness of the pooled effect size. Publication bias was assessed using funnel plots and Egger’s regression test. For studies with zero events in both arms, we applied a standard continuity correction of 0.5 (0.1, 0.01) to both arms, in accordance with Cochrane Handbook recommendations, to facilitate the calculation of RR. Risk Ratio (RR) with 95% confidence interval (CI) was used to compare treatment effects for categorical endpoints. Statistical significance was evaluated using two-sided tests with a *p* < 0.05. Review Manager 5.3 software (Cochrane Collaboration, Oxford, UK) and Stata software version 15.0 (Stata Corporation, College Station, TX, United States) were used for statistical analysis.

## Results

### Literature search results

**Figure 1:**
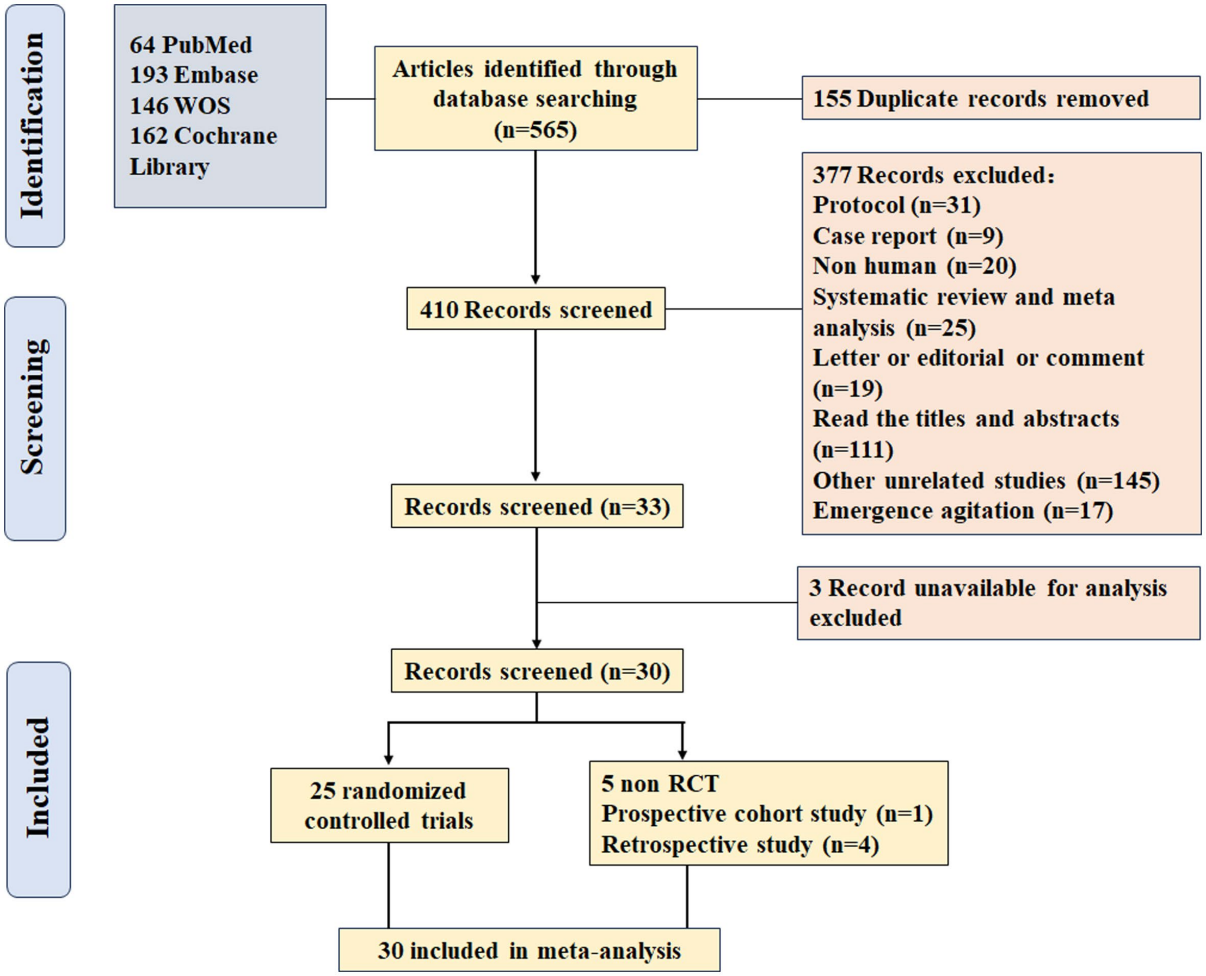
PRISMA flow diagram of the systematic literature search and study selection.

A total of 565 records were identified from the electronic database search (PubMed, Embase, Web of Science, and Cochrane Library). There were 155 duplicated records which were removed using EndNote. 410 unique records underwent title and abstract screening, resulting in the exclusion of 377 publications. The remaining 33 full-text articles were retrieved and assessed for eligibility. Three records were excluded due to unavailability of essential outcome data, the incidence of delirium. Ultimately, 30 studies met all inclusion criteria and were incorporated into the systematic review and meta-analysis, comprising 25 RCT (18–42), 4 retrospective studies (43–46) and a prospective cohort study (47) (Figure 1).

### Characteristics of study

The included studies were conducted in China (*n* = 16), followed by Korea (*n* = 7), Japan (*n* = 6), and Europe (*n* = 1). Remimazolam was infused intravenously during induction or maintenance of general anesthesia in 30 studies, while one in spinal anesthesia (remimazolam was also used for intravenous throughout the procedure) (47). Inhalation anaesthetics including sevoflurane and desflurane were used in 9 studies (18, 22, 23, 25, 34, 36, 43, 45, 46) (Table 1). The control drugs used in Non-Remimazolam group included propofol (18, 19, 21, 24, 26–33, 35, 37–45), dexmedetomidine (20, 47), sevoflurane (25, 34, 36, 43, 45, 46), desflurane (43, 45) and normal saline (20, 22, 23) (Table 1).

**Table 1:** Characteristics of included studies.

Study	Design	Region	Sample size				Population	ASA	Surgery type	Anesthesia type	Assessments	Follow-up
			Remimazolam		Non-remimazolam		Age (years)					(days)
Aoki et al. (43)	Prospective cohort study	Japan	78	Induction: undescribed Maintenance: remimazolam was administered continuously	122 (propofol 83, desflurane 16, sevoflurane 23)	Induction: undescribed Maintenance: undescribed	Age>65	II ~ III	Cardiovascular surgery	GA	CAM-ICU, ICDSC	5
Yang et al. (18)	RCT	China	147	Induction: 0.2–0.3 mg/kg Maintenance: adjusting the infusion dose of remimazolam, BIS 40–60	Propofol 153	Induction: 1.0–1.5 mg/kg Maintenance: adjusting the dose of Propofol, BIS 40–60	Age>60	I ~ III	Orthopedic surgery	GA	CAM	3
Liu et al. (19)	RCT	China	50	Induction: 0.1–0.2 mg/kg Maintenance: TCI of remimazolam 0.4–1.2 mg/kg/h, BIS 40–60	Propofol 50	Induction: 1–2 mg/kg Maintenance: TCI of Propofol 4–10 mg/kg/h, BIS 40–60	Age>65	I ~ III	Colon cancer surgery	GA	CAM-ICU, RASS	7
Kaneko et al. (44)	Retrospective exploratory study	Japan	40	Induction: 3 mg/kg/h and reduced to 0.5 mg/kg/h after loss of consciousness Maintenance: TCI of remimazolam 0.5–0.7 mg/kg/h, BIS 40–60	Propofol 58	Induction: 1–2 mg/kg Maintenance: TCI of Propofol 3–5 mg/kg/h, BIS 40–60	Age>65	Unclear	TAVI	GA	CAM-ICU, CAM	3
Fujimoto et al. (45)	Retrospective observational study	Japan	54	Induction: 12 mg/kg/h Maintenance: TCI of remimazolam 0.5–1 mg/kg/h	176 (propofol 13, desflurane 65, sevoflurane 98)	Induction: 1–2 mg/kg Maintenance: TCI of Propofol 3–5 mg/kg/h or 3–5% desflurane or 0.8–1.5% sevoflurane	Age>65	I ~ III	Proximal femoral fractures	GA	CAM	3
Liao et al. (20)	RCT	China	34	Induction: 0.2 mg/kg Maintenance: TCI of remimazolam 0.3–0.5 mg/kg/h, BIS 40–60	70 (Dexmedetomidine 35, saline 35)	Induction: Dexmedetomidine 0.5ug/kg or saline 5 mL Maintenance: Dexmedetomidine 0.3–0.5 ug/kg/h or saline 0.3–0.5 mL/kg/h, BIS 40–60	Age>65	II ~ III	Gastric cancer	GA	MMSE, MoCA	7
Mao et al. (21)	RCT	China	64	Induction: 0.2–0.3 mg/kg Maintenance: TCI of remimazolam 1–2 mg/kg/h, BIS 40–60	Propofol 64	Induction: 2-3 mg/kg Maintenance: TCI of Propofol 4–10 mg/kg/h, BIS 40–60	84 > Age>18	I ~ III	Urologic surgery	GA	Nu-DESC	3
Yang et al. (22)	RCT	China	51	Induction: 0.2 mg/kg Maintenance: remimazolam 0.2 mg/kg diluted in 10 mL saline, continuous infusion	saline 50	Induction: 2 mg/kg Maintenance: An equivalent volume of saline.	7 > Age>3	I ~ II	Tonsillectomy and adenoidectomy	GA	PAED	3
Cai et al. (23)	RCT	China	79	Induction: 0.2 mg/kg Maintenance: continuous infusion of remimazolam at 1 mg/kg/h, BIS 40–60.	saline 40	Induction: 2–2.5 mg/kg Maintenance: continous infusion of saline at 1 mL/kg/h, BIS 40–60	6 > Age>1	I ~ II	Laparoscopic inguinal hernia repair	GA	PAED, FLACC	1
Fang et al. (24)	RCT	China	364	Induction: 0.2–0.25 mg/kg Maintenance: continuous infusion of remimazolam by BIS 45–60	Propofol 364	Induction: 1.5-2 mg/kg Maintenance: continuous infusion of Propofol by BIS 45–60	90 > Age>60	I ~ III	hip surgery	GA	3D-CAM, CAM-ICU, RASS	3
Lee et al. (47)	Retrospective study	Korea	221	Induction: Spinal anesthesia Maintenance: initial remimazolam at 1–3 mg/kg/h, followed by continuous infusion at 0.5 mg/kg/h	Dexmedetomidine 226	Induction: Spinal anesthesia Maintenance: initial Dexmedetomidine at 1ug/kg over 10 min, followed by at 0.4-1ug/kg/h	Age>65	Unclear	Orthopedic surgery	Spinal anesthesia	CHART-DEL	5
Ryu et al. (25)	RCT	Korea	17	Induction: 12 mg/kg/h Maintenance: continuous infusion of remimazolam by PSI 30–50	sevoflurane 17	Induction: 1.5-2 mg/kg Maintenance: sevoflurane 1.5–2.5% by PSI 30–50	Age>60	I ~ III	Transurethral resection of bladder tumor	GA	CAM	1
Fechner et al. (26)	RCT	Europe	270	Induction: 0.2 mg/kg Maintenance: continuous infusion of remimazolam, Narcotrend 27–60	Propofol 95	Induction: Propofol 2% Maintenance: continuous infusion of Propofol, Narcotrend 27–60	Age>60	III ~ IV	Gastrointestinal, vascular, urological	GA	Nu-DESC	1
Kotani et al. (27)	RCT	Japan	17	Induction: 12 mg/kg/h Maintenance: continuous infusion of remimazolam, SedLine, PSI 25–50	Propofol 18	Induction: 2.5ug/ml Maintenance: continuous infusion of Propofol, SedLine, PSI 25–50	Age>65	unclear	TAVR	GA	CAM-ICU	30
Shimizu et al. (28)	RCT	Japan	33	Induction: 12 mg/kg/h Maintenance: continuous infusion of remimazolam at 1–2 mg/kg/h, BIS 40–60	Propofol 33	Induction: 3–4 ug/ml Maintenance: continuous infusion of Propofol at 2-5ug/ml, BIS 40–60	65 > Age>20	I ~ II	Endoscopic sinus surgery	GA	Undescribed	1
Pan et al. (29)	RCT	China	15	Induction: 0.4 mg/kg Maintenance: continuous infusion of remimazolam at 1 mg/kg/h, BIS 40–60	Propofol 15	Induction: 1.5 mg/kg Maintenance: continuous infusion of Propofol at 4-8 mg/kg/h, BIS 40–60	70 > Age>50	I ~ IV	Endotracheal tumor resection or stent implantation	GA	Undescribed	Undescribed
Zhang et al. (30)	RCT	China	71	Induction: 0.1 mg/kg Maintenance: continuous infusion of remimazolam at 0.3–0.7 mg/kg/h, BIS 40–60	Propofol 71	Induction: 1–1.5 mg/kg Maintenance: continuous infusion of Propofol at 4–10 mg/kg/h, BIS 40–60	66 > Age>46	II ~ III	Intracerebral Surgery	GA	CAM-ICU	90
Zhang et al. (31)	RCT	China	173	Induction: 0.1–0.3 mg/kg Maintenance: continuous infusion of remimazolam by BIS 40–60	Propofol 170	Induction: 1–3 mg/kg Maintenance: continuous infusion of Propofol by BIS 40–60	90 > Age>65	II ~ III	Major abdominal surgery	GA	CAM, CAM-ICU	5
Sim et al. (32)	RCT	Korea	216	Induction: undescribed Maintenance: continuous infusion of remimazolam 1–2 mg/kg/h by BIS 40–60	Propofol 216	Induction: undescribed Maintenance: continuous infusion of Propofol by BIS 40–60	Age>65	I ~ III	Gastrectomy	GA	CAM	3
Cai et al. (33)	RCT	China	68	Induction: 0.2–0.3 mg/kg Maintenance: continuous infusion of remimazolam at 0.3–1.0 mg/kg/h, PSI<50	Propofol 68	Induction: 1–1.5 mg/kg Maintenance: continuous infusion of Propofol at 4-12 mg/kg/h, PSI<50	Age>65	I ~ IV	hip surgery	GA	3D-CAM	3
Kim et al. (46)	Retrospective study	Korea	194	Induction: 6 mg/kg/h Maintenance: continuous infusion of remimazolam at 0.5–2.0 mg/kg/h, BIS 40–60	Sevoflurane 308	Induction: 1–2 mg/kg Maintenance: sevoflurane 1–2.5%, BIS 40–60	Age>65	Unclear	Hip surgery	GA	DSM-5	5
Harimochi et al. (34)	RCT	Japan	28	Induction: 6 mg/kg/h Maintenance: continuous infusion of remimazolam at 1 mg/kg/h, BIS 40–60	Sevoflurane 28	Induction: midazolam 0.05 mg/kg Maintenance: sevoflurane 0.5–1.0%, BIS 40–60	Age>65	III ~ IV	TAVR	GA	CAM-ICU	1
Li et al. (35)	RCT	China	60	Induction: 0.2–0.3 mg/kg Maintenance: continuous infusion of remimazolam at 0.1–0.2 mg/kg/h, BIS 40–60	Propofol 60	Induction: 1.5–2.0 mg/kg Maintenance: propofol 4–6 mg/kg/h, BIS 40–60	85 > Age>65	II ~ III	Spinal surgery	GA	MMSE	7
Lee et al. (36)	RCT	Korea	39	Induction: 0.1 mg/kg Maintenance: continuous infusion of remimazolam at 1–2 mg/kg/h, BIS 30–60	Sevoflurane 39	Induction: 1.5 mg/kg Maintenance: sevoflurane, BIS 30–60	Age>65	I ~ III	Total knee arthroplasty	GA	Undescribed	2
Huang et al. (37)	RCT	China	60	Induction: 0.3 mg/kg Maintenance: continuous infusion of remimazolam at 0.3 mg/kg/h, BIS 40–60	Propofol 60	Induction: 2.0 mg/kg Maintenance: propofol 2 mg/kg/h, BIS 40–60	86 > Age>40	II ~ III	Breast cancer surgery	GA	Nu-DESC	1
Jeon et al. (38)	RCT	Korea	60	Induction: 6 mg/kg/h Maintenance: continuous infusion of remimazolam at 1–2 mg/kg/h, BIS 50	Propofol 62	Induction: 4 ug/ml Maintenance: propofol 2.5–4 ug/ml, BIS 50	80 > Age>65	I ~ III	TURBT and laparoscopic cholecystectomy	GA	DSM-5	1
Lan et al. (39)	RCT	China	73	Induction: 10 mg/kg/h Maintenance: continuous infusion of remimazolam at 0.2–2 mg/kg/h, BIS 40–60	Propofol 73	Induction: 100 mg/min Maintenance: propofol 2.5–4 ug/ml, BIS 40–60	60 > Age>18	I ~ II	Urological surgery	GA	Undescribed	1
Luo et al. (40)	RCT	China	76	Induction: 0.3 mg/kg Maintenance: continuous infusion of remimazolam at 1–3 mg/kg/h, BIS 40–60	Propofol 38	Induction: 2.0–2.5 mg/kg Maintenance: propofol 6–12 mg/kg/h, BIS 40–60	75 > Age>18	I ~ II	Urology, Obstetrics and Gynecology, general surgery, thoracic surgery	GA	MMSE	1
Luo et al. (41)	RCT	China	56	Induction: 0.3 mg/kg Maintenance: continuous infusion of remimazolam at 1–2 mg/kg/h, BIS 40–60	Propofol 56	Induction: propofol 2.0 mg/kg Maintenance: propofol 4–10 mg/kg/h, BIS 40–60	60 > Age>18	I ~ II	Laparoscopic cholecystectomy	GA	Undescribed	1
Lee et al. (42)	RCT	Korea	26	Induction: 6 mg/kg/h Maintenance: continuous infusion of remimazolam at 1–2 mg/kg/h, BIS 40–60	Propofol 27	Induction: propofol 3–4 ug/ml Maintenance: propofol at BIS 40–60	75 > Age>20	Unclear	Radiofrequency catheter ablation of atrial fibrillation	GA	Undescribed	1

Thirteen studies assessed POD using the Confusion Assessment Method (CAM) (18, 25, 32, 44, 45), 3-min diagnostic CAM (3D-CAM) (24, 33) or CAM- Intensive Care Unit (CAM-ICU) (19, 27, 30, 31, 34, 43), and 3 studies (21, 26, 37) used Nursing Delirium Screening Scale (Nu-DESC), 3 (20, 35, 40) used Mini-mental State Examination (MMSE) and Montreal Cognitive Assessment (MoCA). Pediatric Anesthesia Emergence Delirium Scale (PAED) was used for incidence of emergence delirium in 2 studies of child (22, 23). Two studies (19, 24) used Richmond Agitation-Sedation Scale (RASS) and 1 study (47) used CHART-DEL. Uncertain assessment were used in 6 studies (28, 29, 36, 39, 42, 48).

### Risk of bias assessment

**Figure 2:**
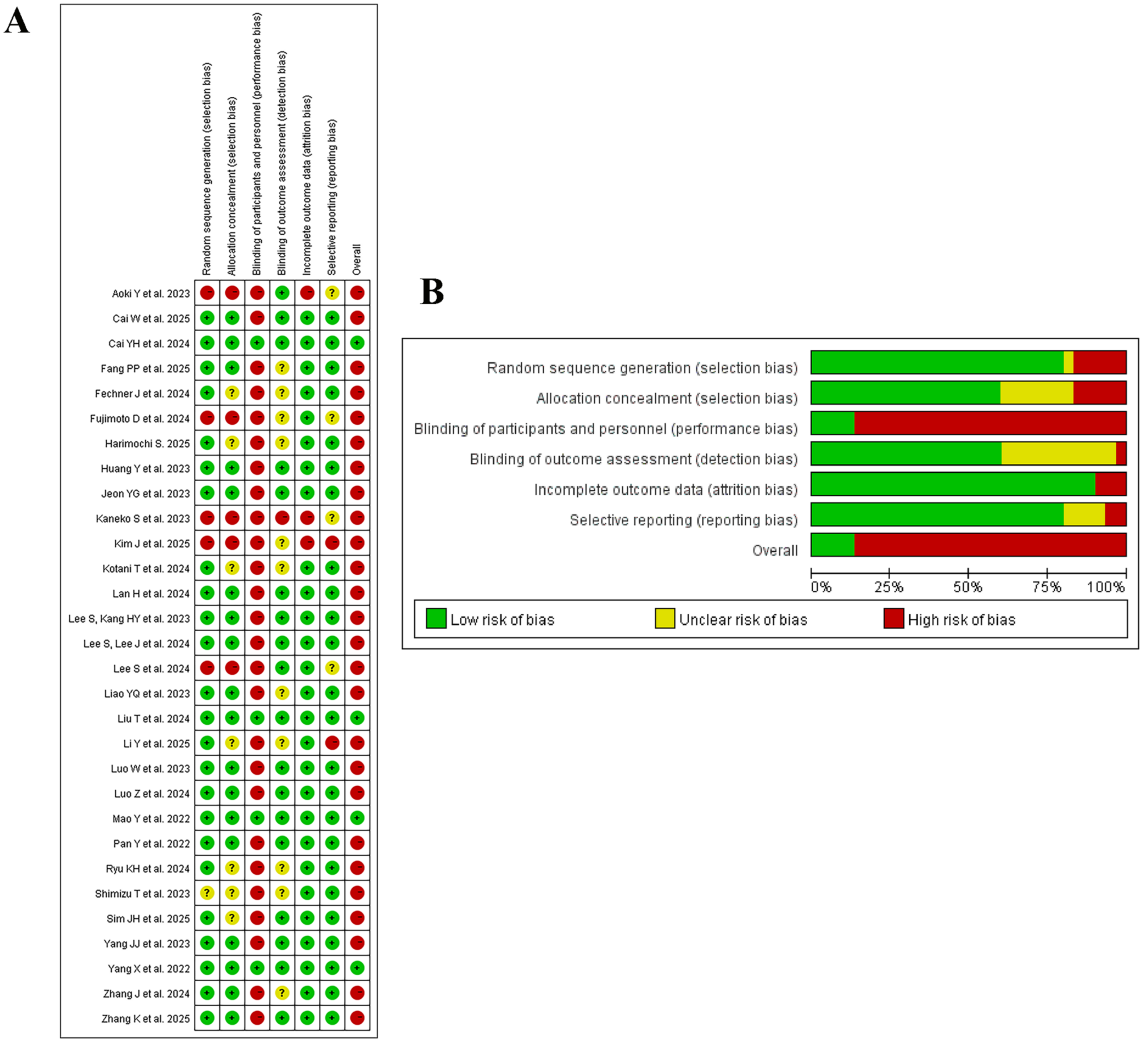
Risk of Bias Assessment of Included Studies. **(A)** Risk of bias for each included study across six domains, presented as a traffic light plot. **(B)** Overall risk of bias across all studies, presented as a summary bar plot. In both plots, green, yellow, and red indicate low, unclear, and high risk of bias, respectively.

The risks of bias for the included studies are shown in Figure 2. Seven trials (25–28, 32, 34, 35) were found to have an unclear risk of selection bias. In the blinding of outcome assessment, 8 studies (20, 24–28, 30, 45) were deemed unclear and 1 studies (27) were deemed high risk. Regarding blinding of patients and personnel, most studies were rated high risk because the anesthesiologists were not blinded. For example, remimazolam and propofol exhibit distinct visual profile. The overall risk of bias for each study was rated as high if any single domain was judged as high risk. This stringent criterion is a primary reason for the high proportion of studies classified as high risk in the present meta-analysis. 4 studies (19, 21–23) showed low risk of bias for overall bias assessment (Figure 2). To empirically assess the influence of study quality, we performed a post-hoc sensitivity analysis excluding studies with a high overall risk of bias. The results of this analysis (pooled RR = 0.50, 95% CI: 0.19–1.31) were consistent with the primary analysis, suggesting that the overall finding of no significant difference in delirium risk was robust to the exclusion of higher risk studies (Supplementary data). This provides some reassurance that the main conclusion is not solely driven by methodologically weaker studies.

### Primary outcome

**Figure 3:**
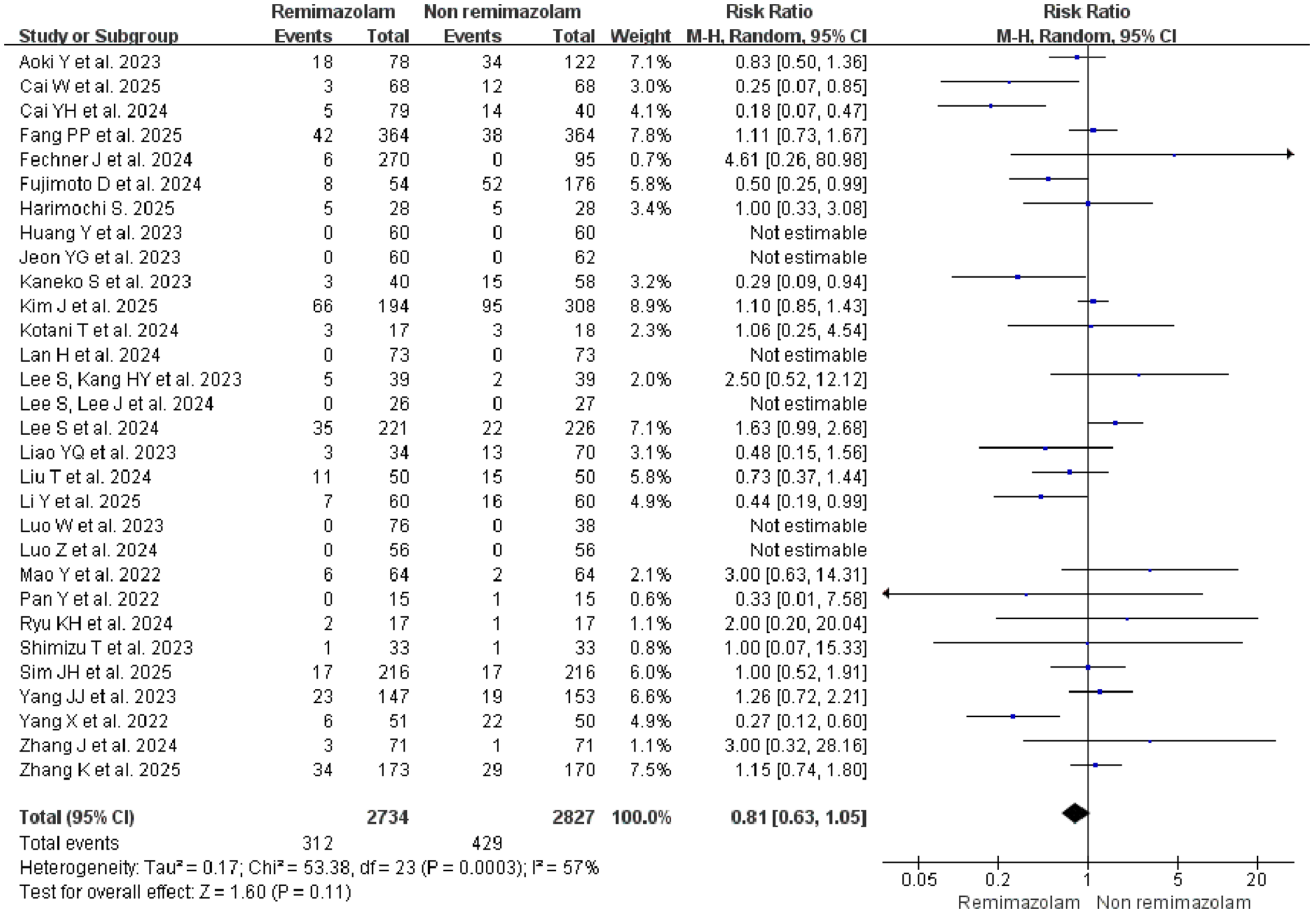
Forest plot for the primary outcome: incidence of delirium (postoperative and emergence delirium combined) between remimazolam and non-remimazolam groups.

The 30 studies randomised a total of 5,561 patients aged from 1 to 90 years old for the analysis of the primary outcome. The overall incidence of delirium (POD and ED together) was 11.4% (312/2734) in the Remimazolam group and 15.2% (429/2827) in the non-Remimazolam group, with no significant difference between groups (Relative Risk (RR) = 0.81, 95% confidence interval (CI), 0.63–1.05, *p* = 0.11) (Figure 3). Subgroup analysis by anesthesia type, however, revealed a significant effect modification. In patients undergoing general anesthesia, remimazolam was associated with a 23% reduction in the risk of delirium (RR = 0.77, 95% CI: 0.60–1.00, *p* = 0.05) (Supplementary Figure S1). For six studies (37–42) with zero events in both arms, a continuity correction of 0.5 (RR = 0.82, 95%, 0.65–1.04, *p* = 0.101) was applied to handle zero-event studies. Sensitivity analyses using alternative correction values 0.1 (RR = 0.82, 95%, 0.65–1.04, *p* = 0.101) and 0.01 (RR = 0.82, 95%, 0.65–1.04, *p* = 0.101) yielded consistent results, confirming the robustness of our findings (Supplementary data).

Given the high heterogeneity among studies (*I*^2^ = 57%, *p* = 0.0003), univariate a meta-regression was subsequently performed to explore potential sources of heterogeneity, including country, study type (RCT or non RCT), age, surgery type, control type (comparators), type of delirium (POD or ED), anesthesia type (GA or spinal anesthesia), assessment of delirium, depth of anesthesia monitor, and the use of flumazenil (Supplementary data). The type of anesthesia was the strongest and only significant modifier of the treatment effect. It explained 93.73% of the between-study variance (adjusted *R^2^* = 93.73, 95% CI: −0.08-1.37, *p* = 0.079). The type of delirium (emergence vs. postoperative) also accounted for a substantial proportion of heterogeneity (adjusted *R^2^* = 47.98%), although this did not reach statistical significance (*p* = 0.31). No other covariates significantly contributed to the observed heterogeneity (Supplementary data).

Subgroup analysis by anesthesia type revealed a significant effect modification. In patients undergoing general anesthesia, remimazolam was associated with a 23% reduction in the risk of delirium (RR = 0.77, 95% CI: 0.60–1.00, *p* = 0.05) (Supplementary Figure S1). A multivariable meta-regression was performed to assess the independent contributions of anesthesia type and delirium type to the observed heterogeneity (Supplementary data). The model demonstrated a perfect fit, explaining 100% of the between-study variance (adjusted *R^2^* = 100%). Variance inflation factors (VIF) for the covariates were all below 1.5, confirming the absence of significant multicollinearity in the model (Supplementary data). A leave-one-out sensitivity analysis was performed to assess the influence of each individual study on the pooled risk ratio. The analysis demonstrated that the overall estimate was robust, as the exclusion of any single study did not substantially alter the pooled result (Supplementary data).

Publication bias was assessed using multiple methods. Egger’s linear regression test yielded a non-significant intercept of 0.16 (95% CI: −0.89-1.22, *p* = 0.749), indicating a low likelihood of small-study effects. Visual inspection of both the funnel plot and the Egger’s regression plot revealed no substantial asymmetry, with studies distributed relatively evenly around the pooled effect estimate. Collectively, these findings suggest that the results of this meta-analysis are unlikely to be substantially influenced by publication bias (Supplementary data).

### Subgroup analyses

#### The type of delirium

**Figure 4:**
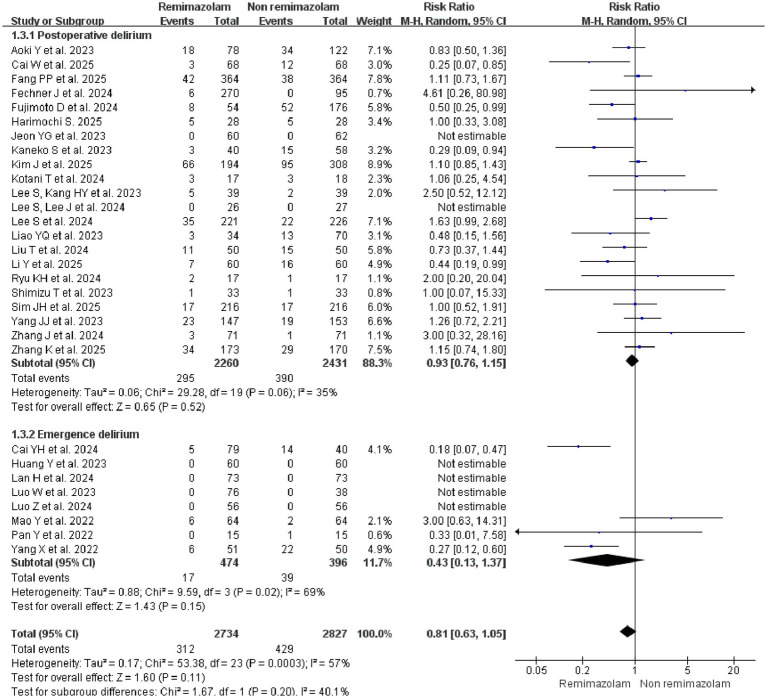
Subgroup analysis: forest plot for the incidence of postoperative delirium and emergence delirium between remimazolam and non-remimazolam groups.

Given that the type of delirium (emergence vs. postoperative) explained a substantial proportion of the between-study heterogeneity (adjusted *R^2^* = 47.98%) in the univariable meta-regression, we performed a subgroup analysis to further explore this potential effect modification. Emergence delirium (ED) and postoperative delirium (POD), while both representing acute perioperative cognitive dysfunction, differ primarily in their temporal profiles. ED is an immediate post-anesthesia phenomenon occurring in the PACU, in contrast to POD, which manifests days following surgery. To ensure nosological precision, studies that reported only “emergence agitation,” a term often used interchangeably and imprecisely with ED (49), were excluded *a priori* during the literature screening phase. No significant difference was observed in 22 studies (18–20, 24–28, 30–36, 38, 42–47) evaluating the incidence of POD between Remimazolam group (13.05%, 295/2260) and Non-Remimazolam group (16.04%, 390/2431) (RR = 0.93; 95% CI, 0.76–1.15; *I*^2^ = 35%; *p* = 0.52), either in 8 studies (21–23, 29, 37, 39–41) on the incidence of emergence delirium (RR = 0.43; 95% CI, 0.13–1.37; *I*^2^ = 69%; *p* = 0.15) (Figure 4).

#### Surgery type

**Figure 5:**
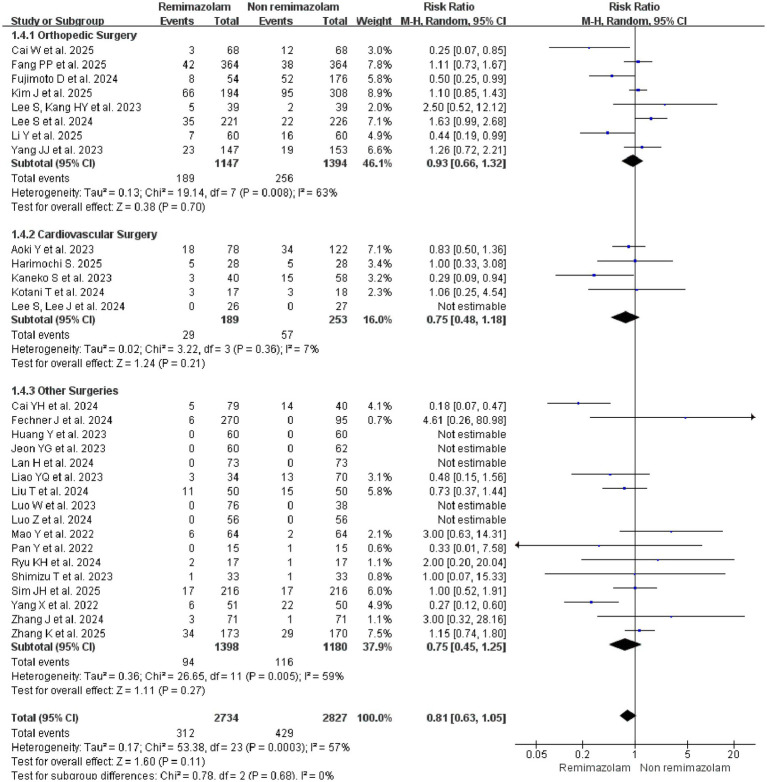
Subgroup analysis: forest plot for the incidence of delirium between remimazolam and non-remimazolam groups, stratified by surgical type.

Subgroup analysis by surgical type revealed no statistically significant reduction in delirium risk for any subgroup: cardiovascular surgery (27, 34, 42–44) (RR = 0.75, 95% CI: 0.48–1.18; *I*^2^ = 7%; *p* = 0.21), orthopedic surgery (18, 24, 33, 35, 36, 45–47) (RR = 0.93, 95% CI: 0.66–1.32; *I*^2^ = 63%; *p* = 0.70), and other surgeries (19–23, 25, 26, 28–32, 37–41) include general surgery, urological surgery, breast cancer surgery, and so on (RR = 0.75, 95% CI: 0.45–1.25; *I*^2^ = 59%; *p* = 0.27) (Figure 5).

#### Remimazolam vs. propofol

**Figure 6:**
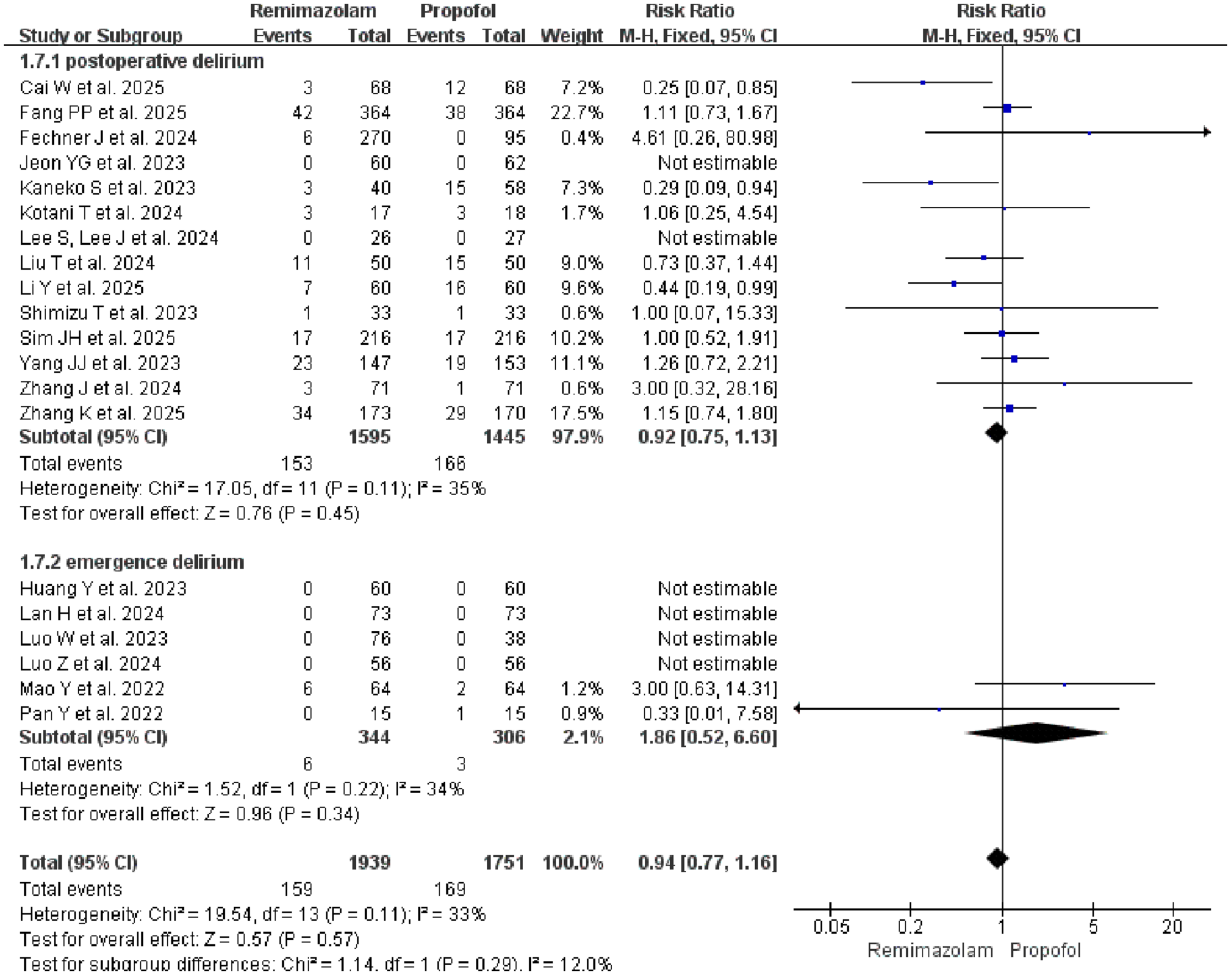
Subgroup analysis: forest plot for the incidence of delirium (postoperative and emergence) in patients receiving remimazolam versus propofol.

A total of 20 studies (18, 19, 21, 24, 26–33, 35, 37–42, 44) comparing the delirium risk of Remimazolam and Propofol were included. The pooled analysis showed no significant difference in the overall delirium risk between the two groups (RR = 0.94, 95% CI: 0.77–1.16; *p* = 0.57; *I*^2^ = 33%). In the subgroup of postoperative delirium, there was no significant difference in risk between Remimazolam and Propofol (RR = 0.92, 95% CI: 0.75–1.13; *p* = 0.45; *I*^2^ = 35%). In the subgroup of emergence delirium, only 2 of the 6 studies reported events that allowed for effect estimation, resulting in extremely limited evidence (Figure 6).

#### Depth of anesthesia

**Figure 7:**
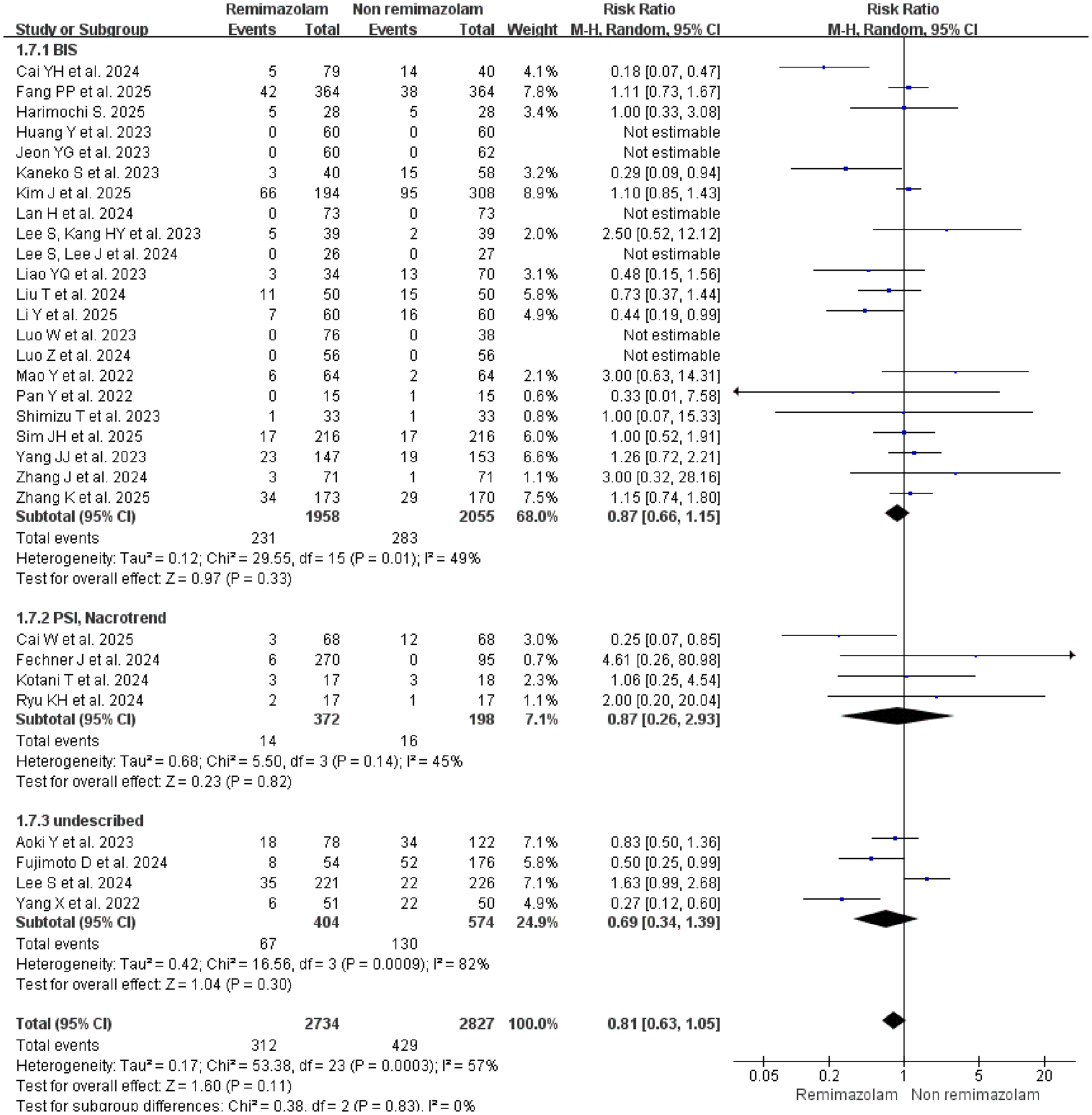
Subgroup analysis: forest plot for the incidence of delirium in patients receiving remimazolam versus non-remimazolam anesthetics, stratified by the method of anesthetic depth monitoring.

Subgroup analysis based on the method of anesthetic depth monitoring showed no statistically significant difference in delirium risk between Remimazolam and non-Remimazolam group, regardless of monitoring type: 4 studies (22, 43, 45, 47) with undescribed monitoring (RR = 0.69, 95% CI: 0.34–1.39, *p* = 0.30; *I*^2^ = 82%), 22 studies (18, 20, 21, 23, 24, 28–32, 34–42, 44, 46, 50) with BIS monitoring (RR = 0.87, 95% CI: 0.66–1.15, *p* = 0.33; *I*^2^ = 49%), and 4 studies (25–27, 33) with PSI/Narcotrend monitoring (RR = 0.87, 95% CI: 0.26–2.93, *p* = 0.82; *I*^2^ = 45%) (Figure 7).

#### Age

**Figure 8:**
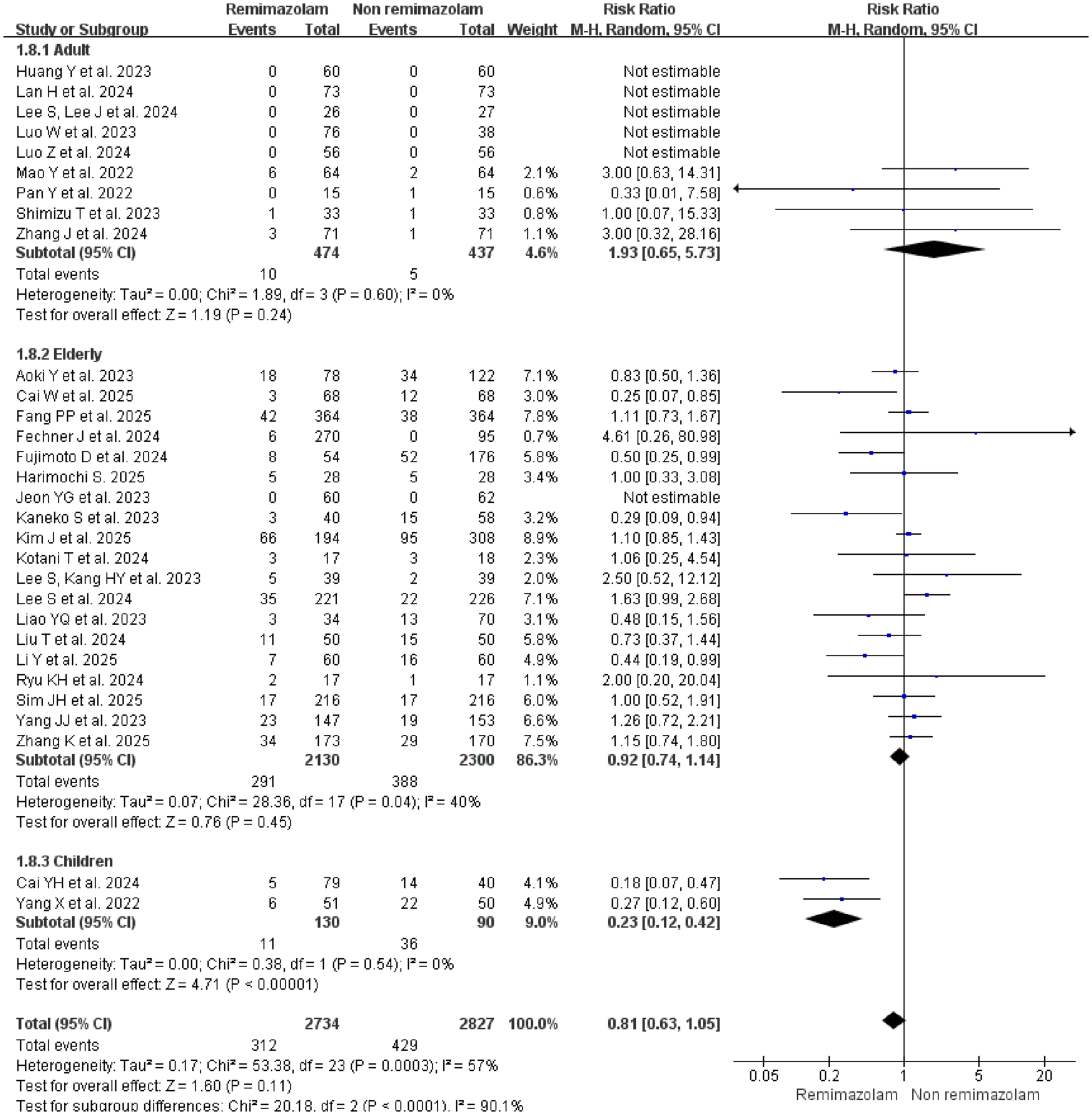
Subgroup analysis: forest plot for the incidence of delirium in patients receiving remimazolam versus non-remimazolam anesthetics, stratified by patient age category.

The inclusion of younger patients may lead to dilution of the effects due to the lower incidence of delirium among younger patients. Subgroup analysis by patient age revealed a significant differential effect. In pediatric patients (22, 23), remimazolam was associated with a 77% reduction in the risk of emergence delirium (RR = 0.23, 95% CI: 0.12–0.42; *p* < 0.00001; *I*^2^ = 0%). In contrast, an analysis of 19 studies (18–20, 24–27, 31–36, 38, 43–47) involving 4,430 patients aged ≥60 years old found no significant benefit in delirium incidence between the remimazolam (13.7%, 291/2130) and non-Remimazolam groups (16.9%, 388/2300) (RR = 0.92, 95% CI: 0.74–1.14; *I*^2^ = 40%; *p* = 0.45) or adults (21, 28–30, 37, 39–42) (RR = 1.93, 95% CI: 0.65–5.73; *I*^2^ = 0%; *p* = 0.24) (Figure 8).

#### The use of flumazenil for antagonism

Subgroup analysis based on the use of flumazenil showed no statistically significant difference in delirium risk between remimazolam and non-Remimazolam groups, regardless of reversal strategy: YES (refer to studies (25, 28, 29, 32, 34–36, 38, 42, 44, 46) in which flumazenil was routinely administered for antagonism of remimazolam at the end of surgery) (RR = 0.87, 95% CI: 0.60–1.27; *I*^2^ = 28%; *p* = 0.47), NO (Studies were classified into the “No” subgroup (21, 23, 27, 40, 41, 45) if the original literature explicitly stated that flumazenil was not administered for routine antagonism at the end of surgery, as well as those where flumazenil was reserved for use only under specific circumstances, such as significantly prolonged emergence time.) (RR = 0.63, 95% CI: 0.23–1.74; *I*^2^ = 71%; *p* = 0.37), and Undescribed (18–20, 22, 24, 26, 30, 31, 33, 37, 39, 43, 47), comprised studies in which the original literature did not report or specify the use of flumazenil (RR = 0.88, 95% CI: 0.63–1.24; *I*^2^ = 60%; *p* = 0.47) (Supplementary Figure S2).

#### Delirium assessment tools

A comprehensive subgroup analysis grouping studies by the primary delirium assessment tool used (CAM/CAM-ICU/3D-CAM (18, 19, 24, 25, 27, 30, 32–34, 43–45), MMSE/Nu-DESC/PAED/DMS-5/CHART-DEL (20–23, 26, 35, 37, 38, 40, 46, 47), and undescribed (28, 29, 36, 39, 41, 42)). The point estimates for the RR were consistent in direction and magnitude across the major assessment tool subgroups: RR = 0.88 (95% CI: 0.69–1.12, *p* = 0.31, *I*^2^ = 27%) for the CAM-family tools and RR = 0.70 (95% CI: 0.38–1.28, *p* = 0.25, *I*^2^ = 80%) for the MMSE/Nu-DESC/PAED group. Both confidence intervals include the null value (RR = 1) and substantially overlap, strongly suggest that there is no statistically significant difference in the effect of remimazolam on delirium risk based on the assessment tool used. This indicates that the primary finding of no significant difference between remimazolam and non remimazolam groups is robust to the methodological heterogeneity introduced by different assessment instruments (Supplementary Figure S3).

### Secondary outcomes

#### PONV

The meta-analysis of postoperative nausea and vomiting (PONV) included data from 19 studies (18–22, 25, 26, 31–37, 39–41, 44). The pooled analysis using a fixed-effect model showed that remimazolam was associated with a statistically significant increase in the risk of PONV compared to non-Remimazolam groups (RR = 1.20, 95% CI: 1.02–1.42; *p* = 0.03). Heterogeneity among the studies was low (*I*^2^ = 20%, *p* = 0.22) (Supplementary Figure S4).

#### Respiratory depression after extubation

A meta-analysis of post-extubation respiratory depression included data from 7 studies (18–20, 33, 35, 41, 42). The pooled analysis using a fixed-effect model showed no statistically significant difference in the risk of respiratory depression between the remimazolam and non-Remimazolam groups (RR = 0.96, 95% CI: 0.63–1.44; *p* = 0.84). Heterogeneity among the studies was low (*I*^2^ = 11%, *p* = 0.34) (Supplementary Figure S5).

#### Extubation time

**Figure 9:**
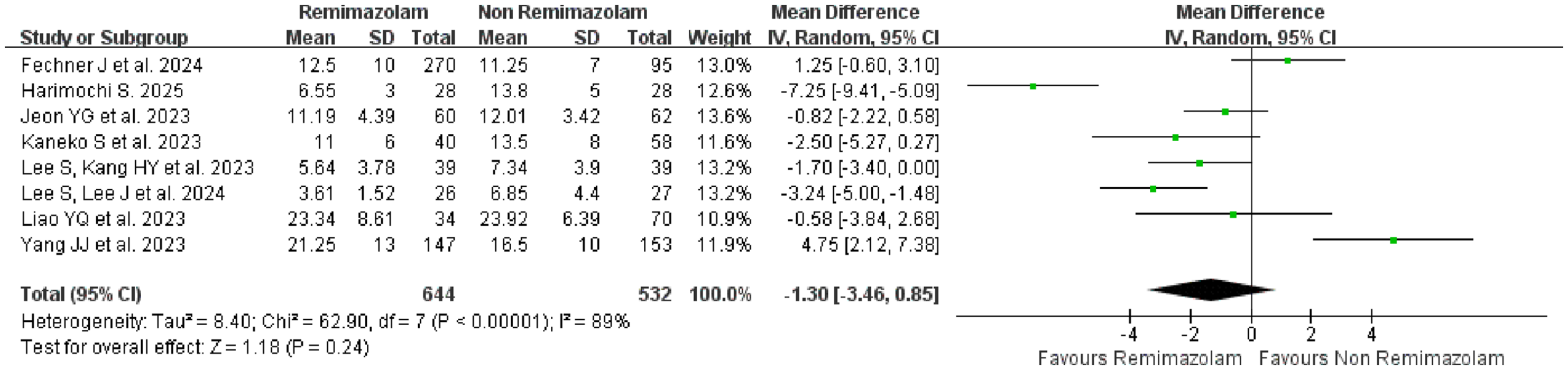
Forest plot for the secondary outcome: extubation time between remimazolam and non-remimazolam groups.

The pooled mean difference was −1.30 (95% CI: −3.46-0.85, *p* = 0.24), indicating no statistically significant difference between remimazolam and non-Remimazolam groups in 8 studies (18, 20, 26, 34, 36, 38, 42, 44). However, extreme heterogeneity was observed (*I*^2^ = 89%, *p* < 0.00001), suggesting substantial inconsistency among the included studies (Figure 9). A leave-one-out sensitivity analysis was performed to assess the influence of individual studies (Supplementary data). The pooled MD ranged from −2.12 to −0.48 when any single study was omitted, with all confidence intervals including zero. The largest change occurred when the study by Harimochi S et al. (34) was excluded, shifting the MD from −1.30 to −0.48, but the conclusion of no statistically significant difference remained unchanged (Supplementary data).

Univariable meta-regression identified two potential sources of heterogeneity (surgical type and the use of flumazenil for reversal). Subgroup analysis by surgical specialty revealed a significant differential effect on extubation time. In cardiovascular surgery, remimazolam was associated with a statistically significant shorter of extubation time compared to non-remimazolam (MD = −4.37, 95% CI: −7.24 to −1.51, *p* = 0.003, *I*^2^ = 80%) (Supplementary Figure S6A). Subgroup analysis by the use of flumazenil for reversal revealed a significant differential effect. In studies that routinely administered flumazenil (Yes subgroup) can significantly shorten extubation time (MD = −3.04, 95% CI: −5.16 to −0.92, *p* = 0.005, *I*^2^ = 84%) (Supplemental Figure S6B).

#### Length of hospital stay

**Figure 10:**
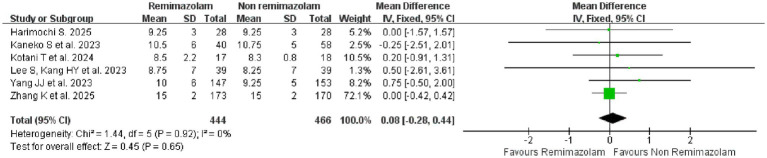
Forest plot for the secondary outcome: hospital length of stay between remimazolam and non-remimazolam groups.

A fixed-effect model meta-analysis of six studies (18, 27, 31, 34, 36, 44) assessing hospital length of stay showed no statistically significant difference between remimazolam and non-remimazolam (MD = 0.08, 95% CI: −0.28-0.44, *p* = 0.65). There was no evidence of heterogeneity among the studies (*I*^2^ = 0%, *p* = 0.92), indicating consistent results across all included trials (Figure 10).

## Discussion

This meta-analysis of 30 clinical trials involving 5,561 patients evaluated the effects of remimazolam on the delirium (POD and ED) compared with other anesthetic agents. The overall analysis found that continuous remimazolam administration was not associated with an increase risk of delirium in patients aged from 1 to 90 years old, especially the risk of delirium in elderly patients. To our knowledge, this is the first study to integrate RCTs and cohort studies comparing continuous remimazolam administration versus non-remimazolam (saline, propofol, dexmedetomidine, sevoflurane, desflurane) on delirium.

Benzodiazepines are widely used as premedication or intraoperative agents in anesthesia protocols due to their sedative and anti-anxiety effects, as well as the ability to minimize intraoperative awareness and anterograde amnesia (51). In recent years, growing concerns have emerged regarding the perioperative use of benzodiazepines, primarily because several evidence suggests that they may be associated with an increased risk of postoperative delirium in elderly patients (52–54). Despite guidelines cautioning against intraoperative benzodiazepines (51, 55), emerging evidence indicates that their administration does not appear to increase POD risk (56). Recently Hao Li et al. (57) found that intraoperative use of midazolam was not associated with an increased risk of postoperative delirium in elderly patients undergoing non cardiac surgery. Current evidence suggests that the association between benzodiazepines and delirium is still controversial.

Remimazolam, a novel ultra-short acting benzodiazepine drug that is independent of hepatic and renal metabolism, can be rapidly hydrolyzed by tissue lipase into inactive metabolites and may reduce the recovery time. However, it has not been explored whether prolonged infusion of remimazolam may increase the risk of POD. Our findings demonstrated equivalent delirium incidence in patients over 60 years of prolonged infusion of remimazolam. Surgical type is one of the risk factors of delirium, particularly for orthopedic (50, 58) and cardiac procedures (6). Although the interaction of surgical stress and sedative pharmacology could theoretically exacerbate cognitive dysfunction, we found equivalent delirium risks with remimazolam in cardiovascular surgery, orthopedic surgery and other surgeries.

The potential biological mechanisms underlying the observed clinical effects of remimazolam, particularly in relation to delirium, warrant a careful and evidence-based discussion. It is crucial to distinguish between mechanisms supported by direct clinical data and those that remain primarily theoretical or derived from preclinical models. The most clinically substantiated mechanism is the superior hemodynamic stability associated with remimazolam compared to other anesthetic agents. Since intraoperative hypotension is a well-established risk factor for postoperative delirium (59, 60), the mitigation of this risk factor represents a plausible and directly supported pathway through which remimazolam could confer a benefit. However, it must be acknowledged that hemodynamic stability is just one of many contributors to delirium, and its relative importance remains unclear.

Other frequently proposed mechanisms, such as anti-inflammatory effects or specific neuroprotection, require a more cautious interpretation. The support for these is primarily derived from animal studies or *in vitro* models (61–63), which demonstrate that remimazolam can attenuate inflammatory cytokine release or reduce neuronal apoptosis under experimental conditions (64–67). However, direct evidence translating these findings to the clinical setting of delirium in humans is currently lacking.

Similarly, while it’s pharmacokinetic profile suggests rapid clearance, which is theoretically beneficial for clear-headed recovery, this does not automatically equate to a reduced incidence of delirium, a complex neuromedical syndrome. The high heterogeneity in our outcomes, along with the null finding regarding flumazenil reversal (which would presumably mitigate any lingering GABAergic effects), suggests that the mechanisms are not fully understood. Therefore, while the hemodynamic advantage is a strong candidate mechanism, the roles of anti-inflammatory and other neuroprotective effects should be presented as compelling hypotheses that require validation through targeted clinical research, such as studies incorporating serial biomarker assessments (e.g., inflammatory markers, neurofilament light chain) in patients receiving remimazolam versus other anesthetics.

Our study found the potential benefit of remimazolam on postoperative ED in the pediatric population where the incidence of emergency delirium can be up to 30% (68). In pediatric populations, continuous infusion or single bolus administration of remimazolam has reduced ED incidence following laparoscopic surgery (23). In children aged 3–6 years, the incidence of ED during induction and maintenance of anesthesia with remimazolam was 8.5% (11/130), significantly lower than the 40% (36/90) observed in the non-remimazolam group (69). Although remimazolam remains off-label for pediatric populations, it has achieved breakthrough progress in this field, with predictable pharmacokinetic properties and safety characteristics (70). Further research is necessary to establish long-term safety, especially long-term neurodevelopmental safety akin to what was obtained in the landmark GAS (71) and PANDA (72) studies, and determine the optimal dosing protocol with potential for broad clinical use in pediatric anesthesia practice.

Numerous observational studies have demonstrated that excessive anesthetic depth, manifested on EEG as burst suppression or persistently low Bispectral Index BIS values, is independent risk factor for delirium (54, 73). However, interventional studies attempting to prevent delirium by titrating anesthetic depth under BIS guidance have yielded inconsistent results (6, 74). We found that among the 22 studies (18–21, 23, 24, 28–32, 34–42, 44, 46) utilizing a BIS target of 40–60 during general anesthesia, there was no significant difference in the incidence of delirium between the remimazolam and non-remimazolam groups. The potential explanation may be that there is a fundamental distinction in the EEG characteristics between remimazolam and other substance such as propofol. Propofol induces a dose-dependent increase in slow-wave and delta-wave activity (75), which correlates linearly with BIS values. In contrast, remimazolam, as a benzodiazepine agent, primarily elicits beta-frequency oscillations (76, 77). This unique pharmacodynamic profile results in a non-linear relationship with conventional anesthesia depth indices, largely calibrated based on propofol data (75). Consequently, at equivalent clinical sedation depths, BIS value under remimazolam anesthesia may be significantly higher (75), creating a risk of underestimating the sedation level if propofol-derived interpretive criteria are applied mechanistically. Future efforts should focus on more refined EEG characteristics, such as frontal alpha power (78, 79) and brain functional connectivity (80, 81), and explore EEG-based individualized closed-loop anesthesia systems, which may represent a more effective approach for prevention.

Flumazenil is widely used to reverse benzodiazepine-induced sedation. Flumazenil competitively antagonizes central GABA receptors, which can reverse sedation within 1–2 min, accelerate wakefulness, further shorten residual sedation time. Consistent with our analysis, routine flumazenil reversal significantly reduced extubation time. However, this advantage did not translate into a reduction in the length of hospital stay. This finding aligns with clinical reality, as hospitalization duration is governed by multiple factors, far beyond the immediate pharmacological effects of the anesthetic reversal agent. However, there is currently no clear evidence to suggest that the use of flumazenil to antagonize remimazolam can reduce the incidence of delirium. Further research is needed to investigate the association between flumazenil antagonism and delirium.

The interpretation of key secondary outcomes, particularly hospital length of stay and mortality, is crucial for a comprehensive understanding of remimazolam’s clinical profile. Our analysis found no statistically significant difference in the length of hospital stay between the remimazolam and non-remimazolam groups (MD = 0.08, 95% CI: −0.28-0.44, *p* = 0.65). Similarly, the available data on mortality were insufficient for a meaningful meta-analysis due to its infrequent reporting in the source literature. The null finding regarding hospital stay should be interpreted with caution. First, the clinical equivalence in this outcome suggests that the potential benefits of remimazolam, such as a faster emergence, may not translate into a shorter overall hospitalization. This is not unexpected, as the duration of hospital stay is a complex endpoint governed by a multitude of factors far beyond the choice of anesthetic agent, including surgical factors (e.g., complexity, complications), patient comorbidities, institutional protocols, and so on. The effect of intraoperative drug choice is likely diluted within this intricate web of determinants. Therefore, the absence of a difference should not be viewed as a failure of remimazolam, but rather as an indication that its advantages are confined to the immediate perioperative period and do not override the more dominant drivers of prolonged hospitalization. The inability to analyze mortality represents a significant evidence gap in the current literature. The paucity of data on mortality precluded a quantitative synthesis. Specifically, the event data were available from only 6 studies (27, 31, 34, 38, 44, 46), the majority of which (4 out of 6) had zero events in both arms (27, 34, 38, 44), thus providing insufficient data for a meaningful pooled estimate. This gap underscores that the existing trials were primarily designed and powered to assess short-term efficacy and safety endpoints, not rare but critical outcomes like survival. We recommend that future large-scale, pragmatic randomized controlled trials be specifically designed and powered to assess mortality as a primary patient-centered outcome. Such trials are essential to definitively characterize the long-term risk–benefit profile of remimazolam.

The interpretation of several outcomes in this meta-analysis, particularly those where the 95% confidence interval included the null value (risk ratio of 1), warrants careful consideration of statistical power. For instance, the analysis of delirium yielded a risk ratio of 0.81 with a 95% CI of 0.63–1.05. While this is conventionally interpreted as ‘no statistically significant difference’, the wide confidence interval that crosses the line of no effect suggests that our result is imprecise. The confidence interval indicates that the true effect could plausibly range from a 37% reduction to a 5% increase in delirium risk. This imprecision leaves open two plausible interpretations: there is truly no clinically important effect of remimazolam on this outcome, or a clinically relevant effect may exist, but our analysis lacked the sufficient statistical power to detect it. The latter possibility, insufficient power, is a common limitation in meta-analyses and could stem from the limited number of studies, small sample sizes within the included trials, or a low baseline event rate for the outcome. Therefore, these particular findings should not be interpreted as definitive evidence of equivalence, but rather as an indication that the current evidence is inconclusive. Future large-scale, well-powered randomized trials are needed to provide a more precise estimate of the effect and to confirm or refute the potential benefits or risks suggested by the point estimates in our analysis.

## Comparative advantages and novel findings with existing meta-analysis

This meta-analysis provides several important advancements over previous systematic reviews (82–84), offering a more nuanced and comprehensive understanding of remimazolam’s perioperative profile. First, our study significantly broadens the clinical generalizability of the evidence. Unlike earlier meta-analyses that were often focused on specific settings (82, 85), we incorporated a wide spectrum of surgical types, including orthopedic, cardiac, urological, and abdominal procedures. While the recent meta-analysis by Li C et al. (84) descriptively assessed the incidence and predictors of delirium with remimazolam, our study provides a direct comparative effectiveness analysis against alternative anesthetics, specifically explores the differential risk between emergence and postoperative delirium. Furthermore, to enhance the relevance for delirium outcomes, we explicitly excluded populations undergoing minor sedation procedures, which have an inherently low delirium risk. This deliberate focus on a more diverse yet clinically homogenous surgical population strengthens the external validity of our findings. Second, we employed a methodologically more rigorous and exploratory approach. While prior studies predominantly compared remimazolam against propofol (60, 82, 85). Our analysis included a broader range of control interventions (e.g., dexmedetomidine, inhaled anesthetics, saline). This enabled a more robust comparative effectiveness assessment. More importantly, we conducted detailed, pre-specified subgroup analyses to dissect procedure-specific and population-specific outcomes. This approach moves beyond an averaged effect to explore potential effect modifiers, a key limitation in previous syntheses. Third, and most notably, our analysis yields a novel and clinically significant finding. Although Wang M et al. (83) included pediatric patients, our study is the first to perform a dedicated subgroup analysis by age. This revealed a significant advantage of remimazolam in reducing the risk of emergence delirium in the pediatric population, a distinct finding that was previously obscured. This represents a tangible contribution to the field, pinpointing a specific patient group that may derive particular benefit from remimazolam, while also highlighting the need for further investigation into its long-term neurodevelopmental safety. By integrating heterogeneous patient cohorts within a refined analytical framework, our work provides a comprehensive and clinically actionable evidence base supporting the strategic use of remimazolam in optimized anesthetic protocols.

## Limitations

Several gaps and challenges in the literature warrant attention. Firstly, a key limitation of our study is the inclusion of both randomized trials and observational studies. While this enhances the generalizability of our findings to broader clinical settings, it introduces a potential for confounding bias inherent in non-randomized designs. However, a sensitivity analysis limited to RCTs produced a nearly identical effect estimate (Supplementary data), which strengthens confidence in the primary conclusion that remimazolam does not significantly increase the risk of delirium compared to other anesthetic regimens. Secondly, outcomes such as mortality were not analyzed, as these were infrequently reported in the source literature. The available data were insufficient for a meaningful meta-analysis on this endpoint. Furthermore, the main limitation of meta-analysis is the significant heterogeneity included in the studies due to different surgical type, age, control group, delirium assessment tools and so on, though results remained consistent. Our search was restricted to studies published in English, potentially excluding relevant data published in other languages, which may introduce language bias. Moving on, the generalizability of our findings is limited by the geographical restriction of the included evidence, almost all studies were conducted in East Asia. This limitation arises from potential variations in genetics, clinical practice, and outcome assessment across regions. Therefore, future multi-regional trials are essential to validate these results in more diverse populations. Finally, the risk of bias assessment indicated that a proportion of the included studies had a high risk of bias, primarily in the domain of blinding of participants and personnel. This is a common and often unavoidable limitation in trials comparing pharmacological interventions with distinct visual profiles (e.g., propofol vs. remimazolam). The potential impact of this performance bias must be carefully considered. A post-hoc sensitivity analysis excluding studies with a high overall risk of bias. The results of this analysis (pooled RR = 0.50, 95% CI: 0.19–1.31) (Supplementary data) were consistent with the primary analysis, suggesting that the overall finding of no significant difference in delirium risk was robust to the exclusion of higher risk studies. This limitation underscores the need for caution when interpreting the results, and emphasizes the complexity of anesthesia practice in various clinical contexts.

## Conclusion

Prolonged intravenous administration of remimazolam during the operation does not increase the risk of delirium. Remimazolam has potential benefits in the pediatric population as it reduces the risk of emergence delirium. Caution may be warranted in drawing conclusions about a causal relationship between remimazolam and delirium, as the occurrence of delirium is also influenced by various factors.

## Data Availability

The original contributions presented in the study are included in the article/Supplementary material, further inquiries can be directed to the corresponding authors.

## Glossary

ASA: American society of anesthesiologists
BIS: Bispectral index
CAM: Confusion assessment method
CAM-ICU: CAM-intensive care unit
CHART-DEL: Confusion health assessment recovery tool—DELirium
CI: confidence interval
3D-CAM: 3-min diagnostic CAM
DSM: Diagnostic and statistical manual of mental disorders
ED: emergence delirium
EEG: Electroencephalogram
GABA_A_: The *γ*-aminobutyric acid type A
ICD: International classification of diseases
MMSE: Mini-mental state examination
MoCA: Montreal cognitive assessment
Nu-DESC: Nursing delirium screening scale
PAED: Pediatric anesthesia emergence delirium scale
PACU: Post anesthesia care unit
POD: postoperative delirium
PONV: postoperative nausea and vomiting
RASS: Richmond agitation-sedation scale
RCT: Randomized controlled trial
RR: risk ratio
TAVI: Transcatheter aortic valve implantation
TAVR: Transcatheter aortic valve replacement
