# Supplementary material for: The delirium dichotomy of remimazolam: a differential risk profile for emergence delirium versus postoperative delirium in surgical patients: a systematic review and meta-analysis
Source: Front Med (Lausanne). 2026 May 11;13:1841225. doi: 10.3389/fmed.2026.1841225 (PMC13199243; doi:10.3389/fmed.2026.1841225)
Supplement: Supplementary file 1 [file Supplementary_file_1.docx]

**Supplementary Figure**

**Supplementary Figure S1.** Subgroup Analysis: Forest Plot for the Incidence of Delirium Between Remimazolam and Non-Remimazolam Groups, Stratified by Anesthesia Type.

**

**

**Supplementary Figure S2.** Subgroup Analysis: Forest Plot for the Incidence of Delirium Between Remimazolam and Non-Remimazolam Groups, Stratified by the Use of Flumazenil Antagonism.

**

**

**Supplementary Figure S3.** Subgroup Analysis: Forest Plot for the Incidence of Delirium Between Remimazolam and Non-Remimazolam Groups, Stratified by the Primary Delirium Assessment Tool Used.

**

**

**Supplementary Figure S4.** Forest Plot for the Secondary Outcome: Risk of Postoperative Nausea and Vomiting (PONV) Between Remimazolam and Non-Remimazolam Groups.

**

**

**Supplementary Figure S5.** Forest Plot for the Secondary Outcome: Risk of Post-Extubation Respiratory Depression Between Remimazolam and Non-Remimazolam Groups.

**

**

**Supplementary Figure S6.** Subgroup Analysis of Factors Influencing Extubation Time: Surgical Specialty and Flumazenil Reversal Between Remimazolam and Non-Remimazolam Groups. A: Stratified by Surgical Specialty.​ B: Stratified by Use of Flumazenil for Reversal.​

**

**

**1. The detailed search strategy for each database is presented.**

**APPENDIX**

**PubMed/MEDLINE**

("remimazolam"[MeSH Terms] OR "remimazolam"[All Fields] OR "ONO 2745"[All Fields] OR "ONO2745"[All Fields] OR "CNS7056"[All Fields] OR "BYFAVO"[All Fields])

AND

("propofol"[MeSH Terms] OR "propofol"[All Fields] OR "dexmedetomidine"[MeSH Terms] OR "dexmedetomidine"[All Fields] OR "sevoflurane"[MeSH Terms] OR "sevoflurane"[All Fields] OR "isoflurane"[MeSH Terms] OR "isoflurane"[All Fields] OR "desflurane"[MeSH Terms] OR "desflurane"[All Fields] OR "Hypnotics and Sedatives"[MeSH Terms] OR "Hypnotics and Sedatives"[All Fields])

AND

("Anesthesia, General"[MeSH Terms] OR "anesthesia"[All Fields] OR "anaesthesia"[All Fields] OR "Anesthesia and Analgesia"[MeSH Terms])

AND

("Delirium"[MeSH Terms] OR "delirium"[All Fields] OR "Postoperative Delirium"[All Fields] OR "Emergence Delirium"[All Fields] OR "Postoperative Cognitive Dysfunction"[MeSH Terms] OR "POD"[All Fields] OR "ED"[All Fields])

AND

("randomized controlled trial"[Publication Type] OR "controlled clinical trial"[Publication Type] OR "clinical trials as topic"[MeSH Terms] OR "trial"[Title] OR random*[Title/Abstract] OR placebo*[Title/Abstract])

**COCHRANE**

("remimazolam" OR "ONO 2745" OR "ONO2745" OR "CNS7056" OR "BYFAVO")

AND

("propofol" OR "dexmedetomidine" OR "sevoflurane" OR "isoflurane" OR "desflurane" OR "Hypnotics and Sedatives")

AND

("Anesthesia, General" OR "anesthesia" OR "anaesthesia" OR "Anesthesia and Analgesia")

AND

("Delirium" OR "delirium" OR "Postoperative Delirium" OR "Emergence Delirium" OR "Postoperative Cognitive Dysfunction" OR "POD" OR "ED")

**EMBASE**

('remimazolam'/exp OR 'remimazolam' OR 'ono 2745'/exp OR 'ono 2745' OR 'ono2745'/exp OR 'ono2745' OR 'cns7056'/exp OR 'cns7056' OR 'byfavo'/exp OR 'byfavo')

AND

('propofol'/exp OR 'propofol' OR 'dexmedetomidine'/exp OR 'dexmedetomidine' OR 'sevoflurane'/exp OR 'sevoflurane' OR 'isoflurane'/exp OR 'isoflurane' OR 'desflurane'/exp OR 'desflurane' OR 'hypnotics and sedatives'/exp OR 'hypnotics and sedatives')

AND

('anesthesia, general'/exp OR 'anesthesia, general' OR 'anesthesia'/exp OR 'anesthesia' OR 'anaesthesia'/exp OR 'anaesthesia' OR 'anesthesia and analgesia'/exp OR 'anesthesia and analgesia')

AND

('delirium'/exp OR 'delirium' OR 'postoperative delirium'/exp OR 'postoperative delirium' OR 'emergence delirium'/exp OR 'emergence delirium' OR 'postoperative cognitive dysfunction'/exp OR 'postoperative cognitive dysfunction' OR 'pod'/exp OR 'pod' OR 'ed')

AND

('randomized controlled trial'/exp OR 'randomized controlled trial' OR 'controlled clinical trial'/exp OR 'controlled clinical trial' OR 'clinical trials'/exp OR 'clinical trials' OR 'trial'/exp OR 'trial')

**WEB OF SCIENCE**

("remimazolam" OR "ONO 2745" OR "ONO2745" OR "CNS7056" OR "BYFAVO")

AND

("propofol" OR "dexmedetomidine" OR "sevoflurane" OR "isoflurane" OR "desflurane" OR "Hypnotics and Sedatives")

AND

("Anesthesia, General" OR "anesthesia" OR "anaesthesia" OR "Anesthesia and Analgesia")

AND

("Delirium" OR "delirium" OR "Postoperative Delirium" OR "Emergence Delirium" OR "Postoperative Cognitive Dysfunction" OR "POD" OR "ED")

AND

("randomized controlled trial" OR "controlled clinical trial" OR "clinical trials" OR "trial")

**2. For six studies with zero events in both arms, a continuity correction was applied to handle zero-event studies**

**2.1 a continuity correction of 0.5 to all cells was applied to handle zero-event studies**


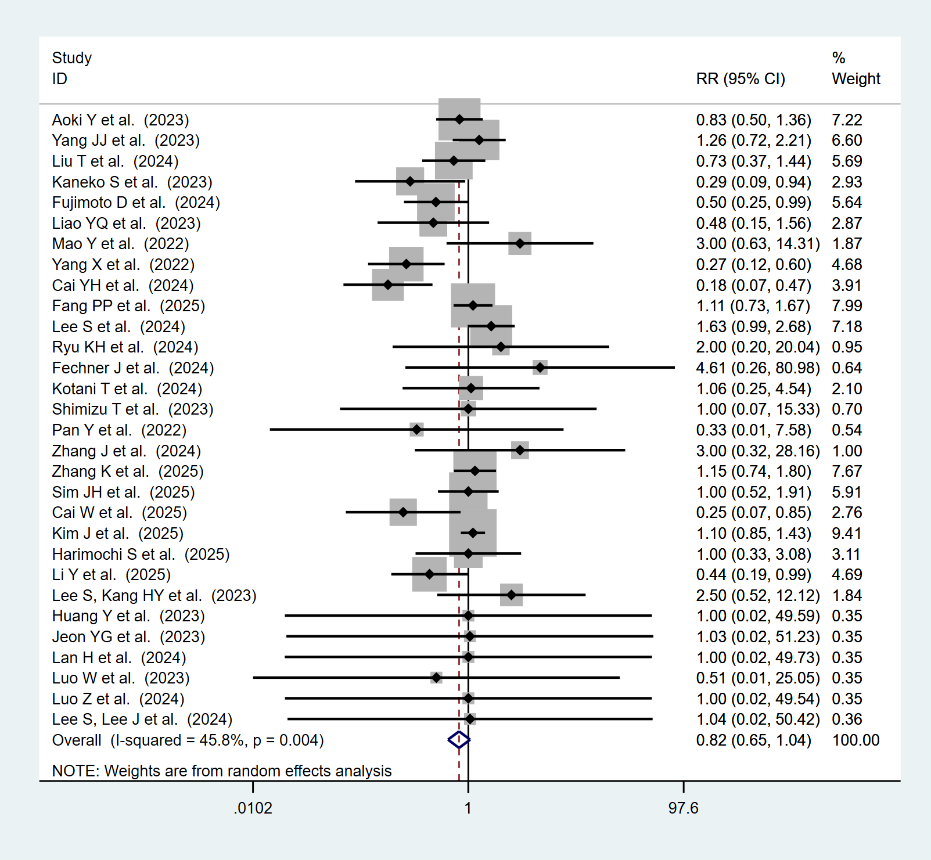


A total of 30 studies were included in the quantitative synthesis. The pooled risk ratio was 0.82 (95% CI: 0.65-1.04, p = 0.101) with low-to-moderate heterogeneity (I² = 45.8%). Sensitivity analysis including studies with zero events in both arms yielded results consistent with the primary analysis, confirming the robustness of the findings.

**2.2 a continuity correction of 0.1 to all cells was applied to handle zero-event studies**


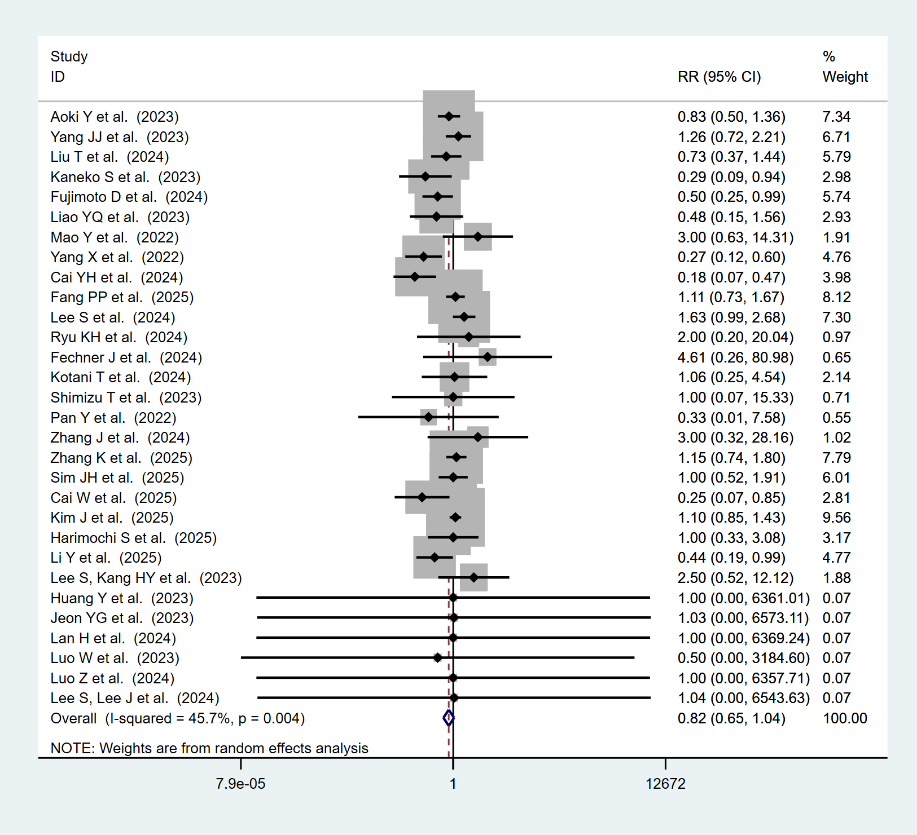


A total of 30 studies were included in the quantitative synthesis. The pooled risk ratio was 0.82 (95% CI: 0.65-1.04, p = 0.101) with low-to-moderate heterogeneity (I² = 45.7%). Sensitivity analysis including studies with zero events in both arms yielded results consistent with the primary analysis, confirming the robustness of the findings.

**2.3 a continuity correction of 0.01 to all cells was applied to handle zero-event studies**


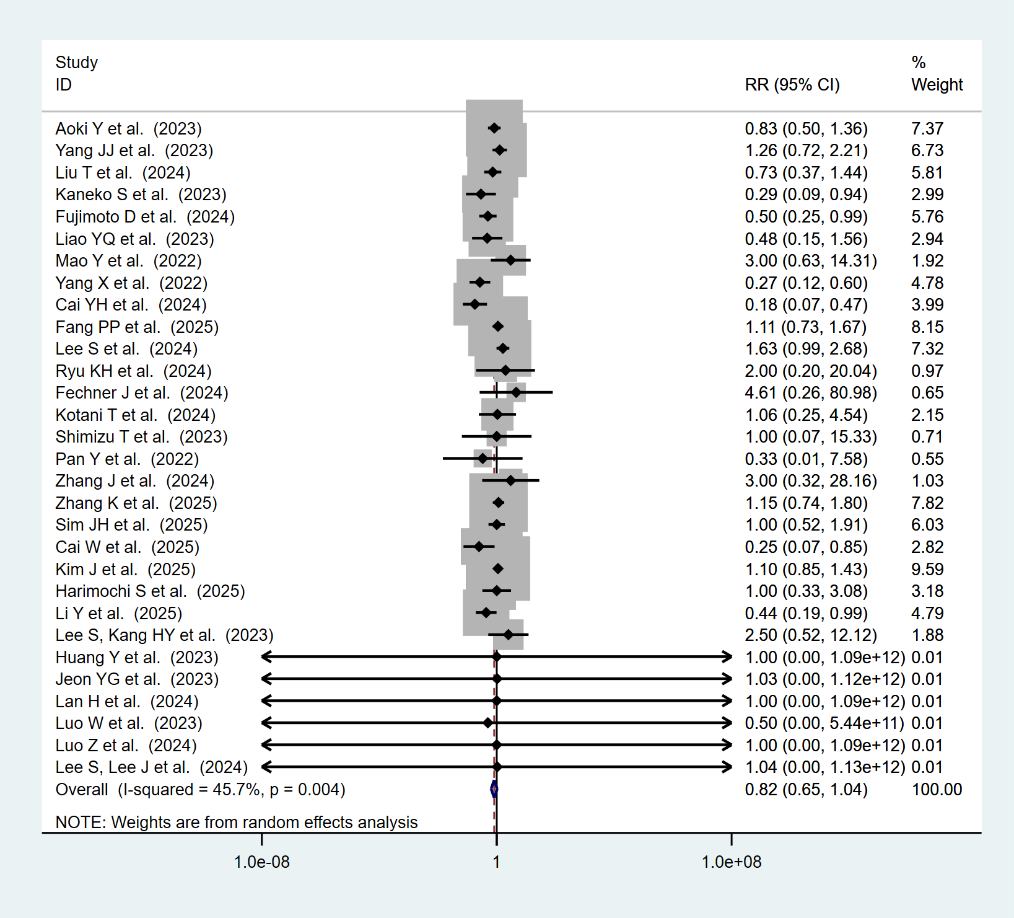


A total of 30 studies were included in the quantitative synthesis. The pooled risk ratio was 0.82 (95% CI: 0.65-1.04, p = 0.101) with low-to-moderate heterogeneity (I² = 45.7%). Sensitivity analysis including studies with zero events in both arms yielded results consistent with the primary analysis, confirming the robustness of the findings.

**3. A meta-regression was performed to explore potential sources of heterogeneity**

**3.1 Country**


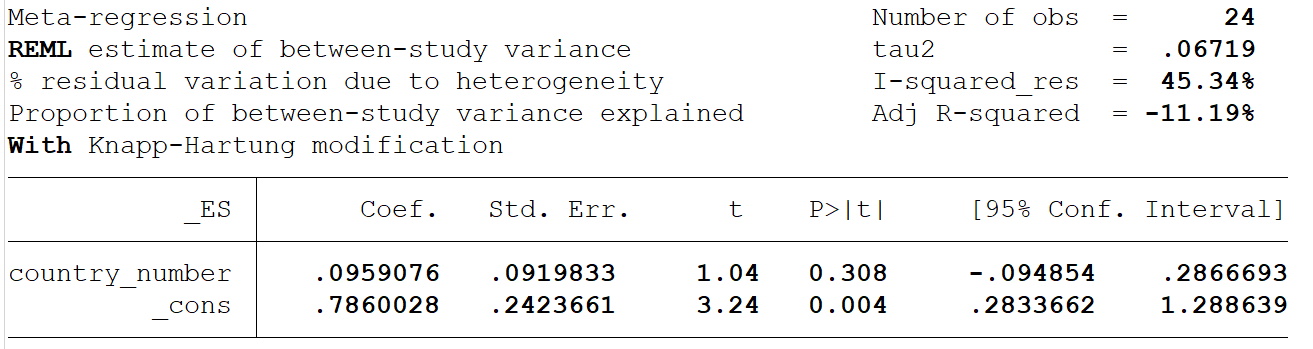


**3.2 study type (RCT or non RCT)**


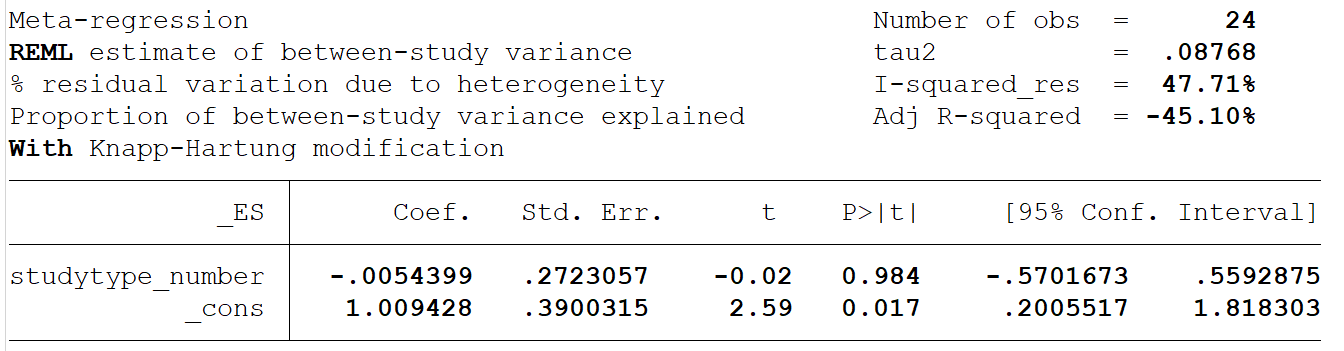


**3.3 age**


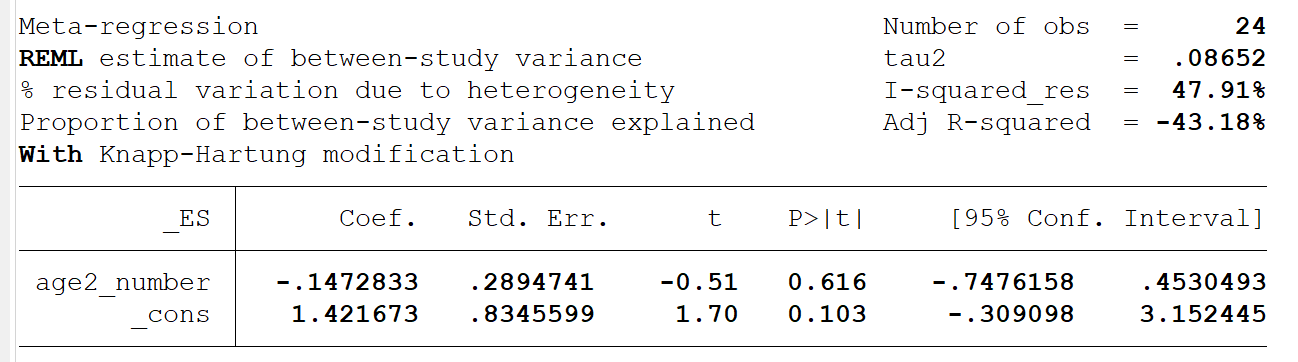


**3.4 surgery type**


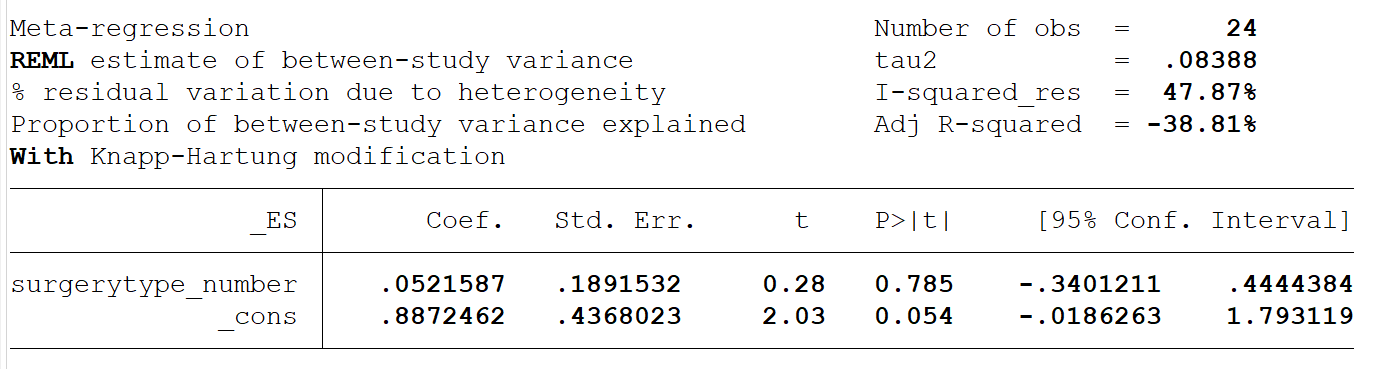


**3.5 control type (comparators)**


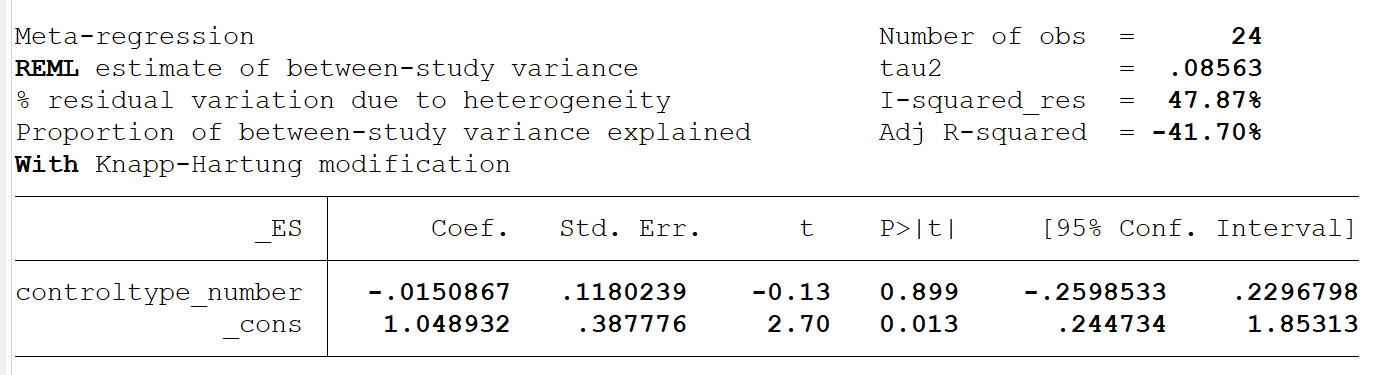


**3.6 types of delirium (POD and ED)**


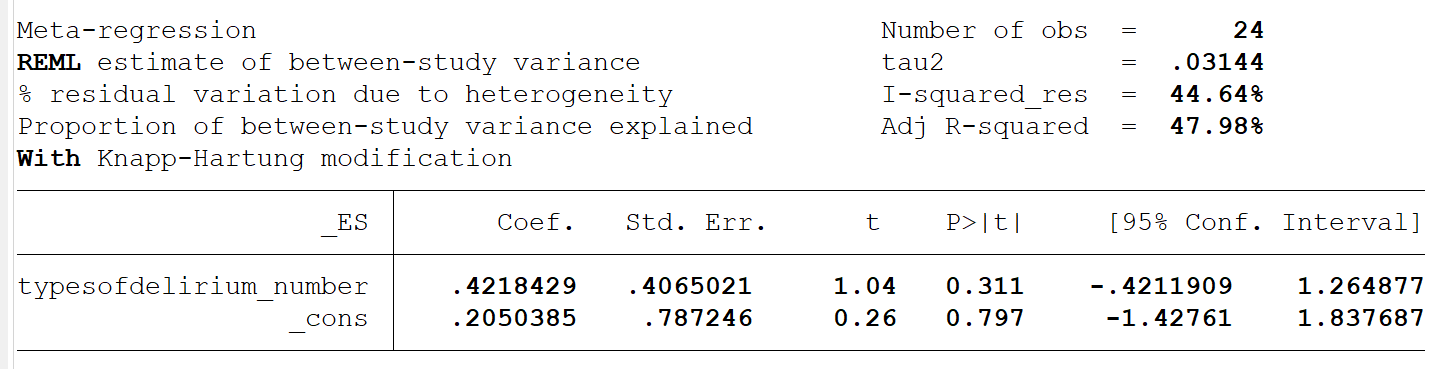


**3.7 anesthesia type (GA or Spinal anesthesia)**


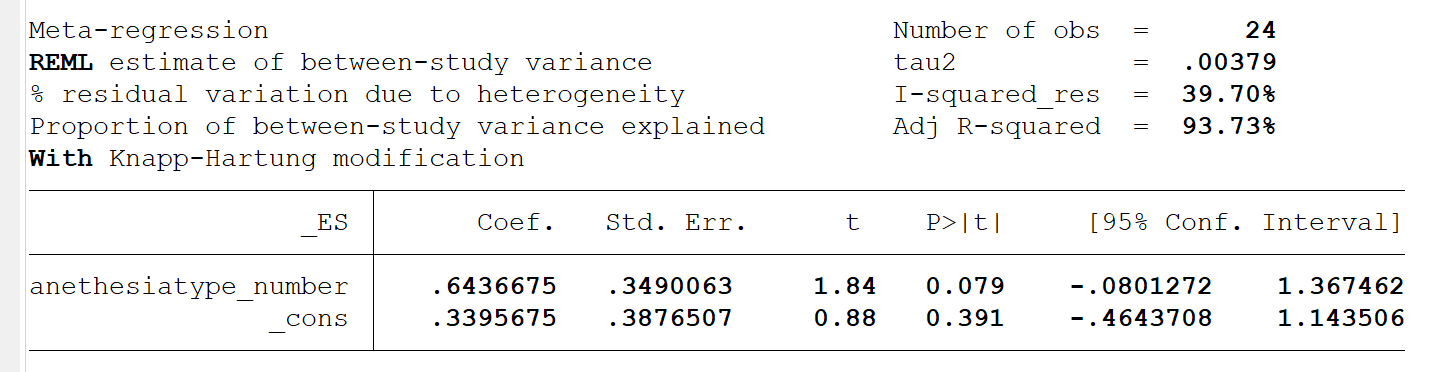


**3.8 assessments of delirium**


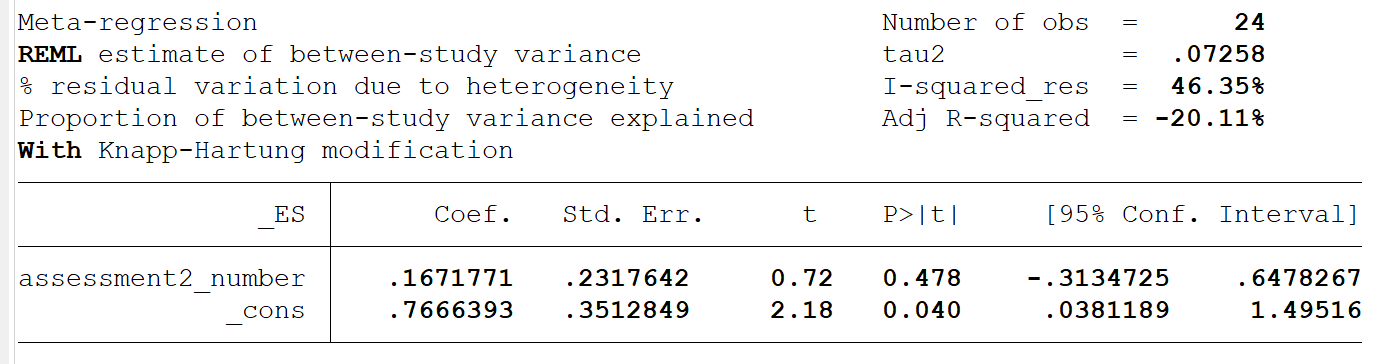


**3.9 depth of anesthesia monitor**


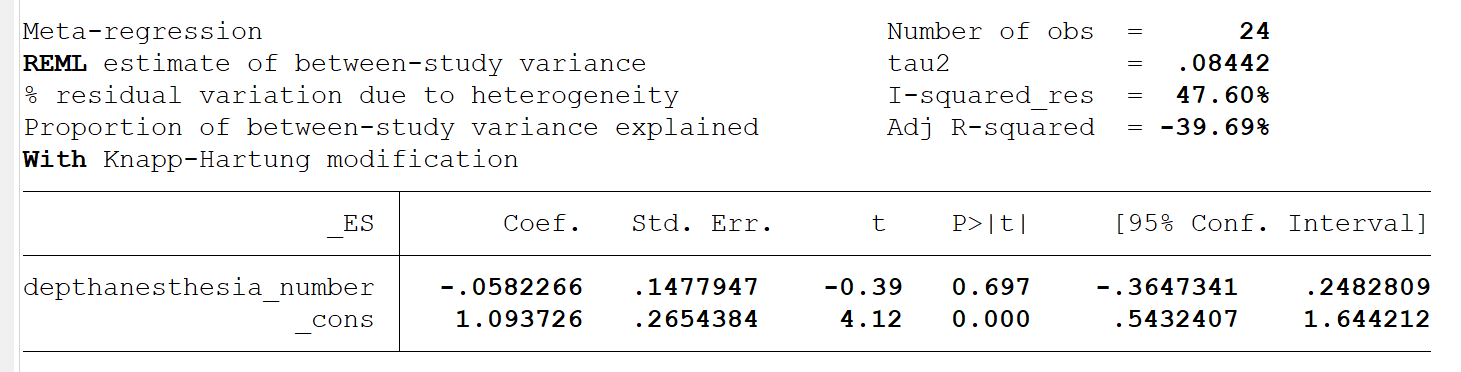


**3.10 the use of flumazenil**


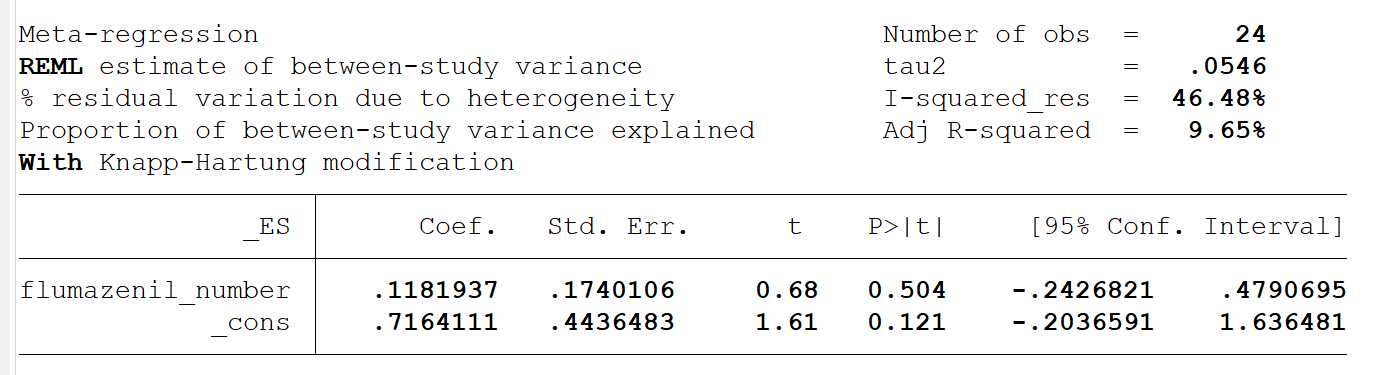

**4. A multivariable meta-regression was performed to assess the independent contributions of anesthesia type and delirium type**


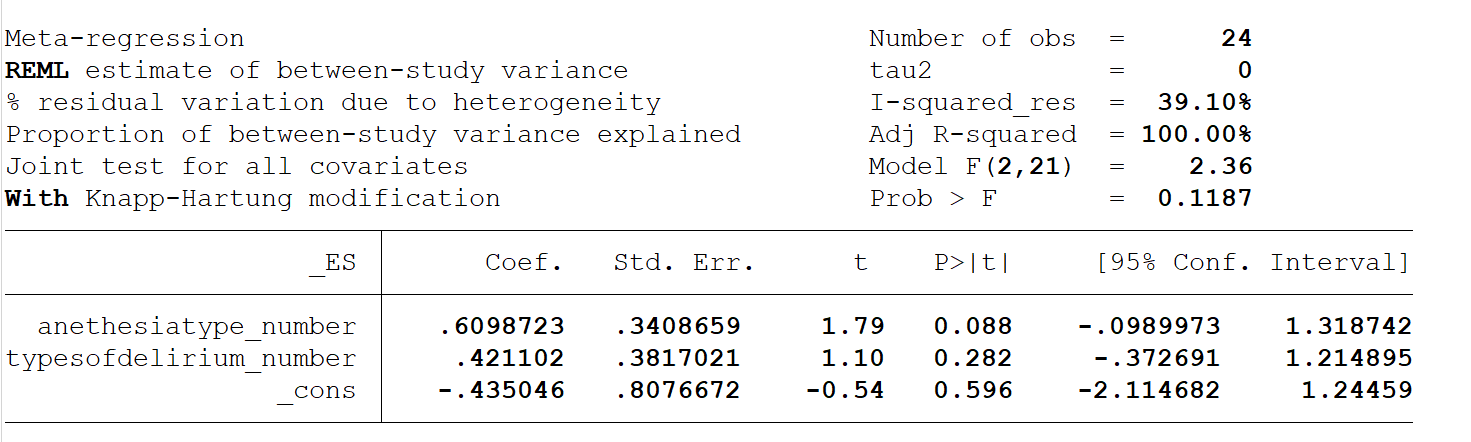


A multivariable meta-regression was performed to assess the independent contributions of anesthesia type and delirium type to the observed heterogeneity. The model demonstrated a perfect fit, explaining 100% of the between-study variance (adjusted R^2^ = 100%). Anesthesia type emerged as a strong, independent predictor of delirium risk, approaching statistical significance (95% CI: -0.10 to 1.32, p = 0.088), corresponding to an 84% increase in risk for certain anesthetic techniques. In contrast, the type of delirium (emergence vs. postoperative) was not a significant predictor after adjusting for anesthesia type (p = 0.282).


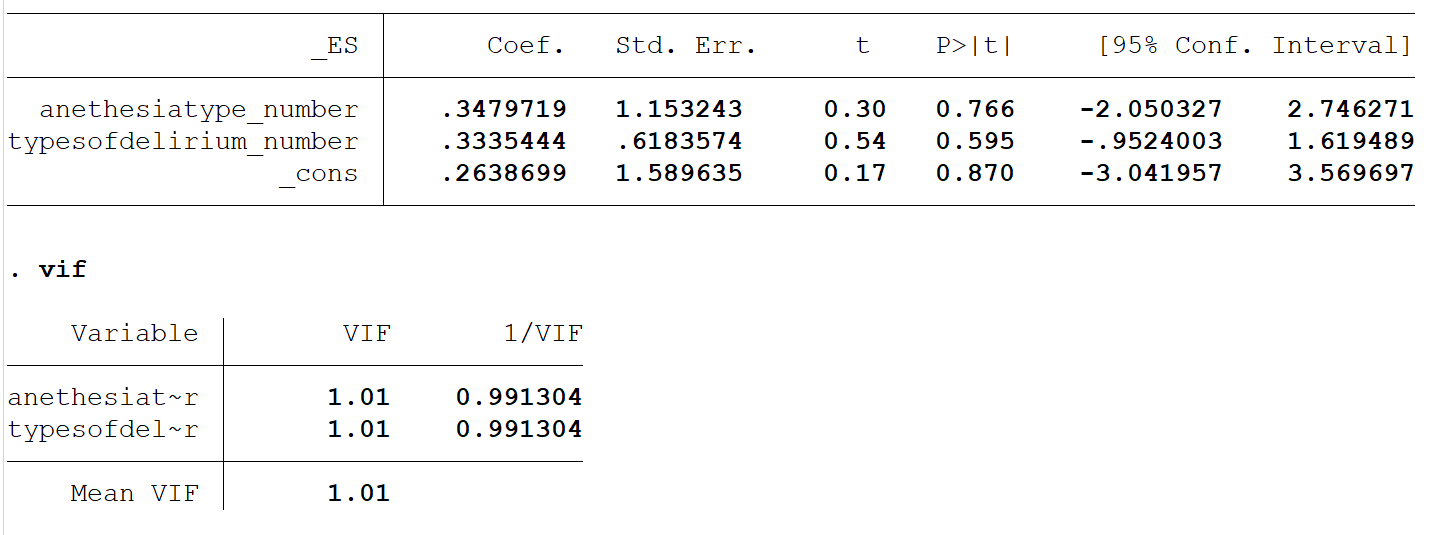


Variance inflation factors (VIF) for the covariates were all below 1.5, confirming the absence of significant multicollinearity in the model.

**5. A leave-one-out sensitivity analysis**

A leave-one-out sensitivity analysis was performed to assess the influence of each individual study on the pooled risk ratio. The analysis demonstrated that the overall estimate was robust, as the exclusion of any single study did not substantially alter the pooled result.

The recalculated risk ratios ranged from 0.79 to 0.83, with all 95% confidence intervals overlapping and including the null value (RR = 1). This confirms that our primary conclusion is not driven by any individual study.

**6. Publication bias**

**6.1 Egger’s linear regression test**


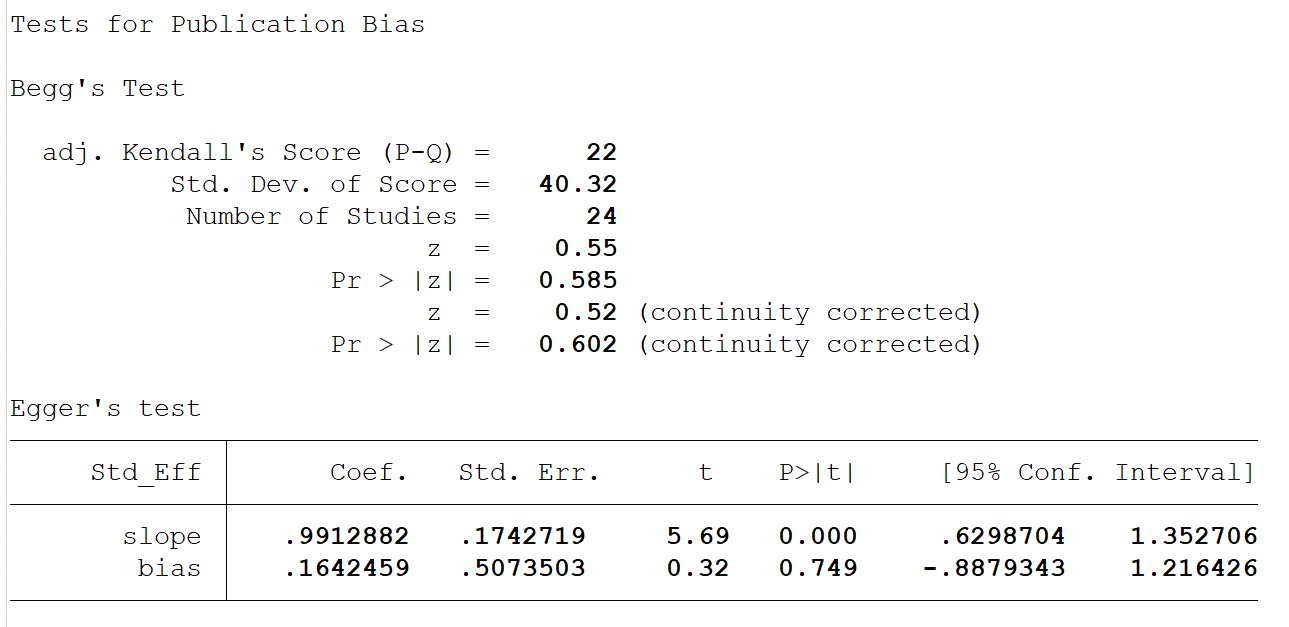


Egger's linear regression test yielded a non-significant intercept of 0.16 (95% CI: -0.89 to 1.22, P = 0.749), indicating a low likelihood of small-study effects.

**6.2 the funnel plot**

Visual inspection of both the funnel plot and the Egger's regression plot revealed no substantial asymmetry, with studies distributed relatively evenly around the pooled effect estimate. Collectively, these findings suggest that the results of this meta-analysis are unlikely to be substantially influenced by publication bias.

**7. A leave-one-out sensitivity analysis was performed for extubation time**

A leave-one-out sensitivity analysis was performed to assess the influence of individual studies. The pooled MD ranged from -2.12 to -0.48 when any single study was omitted, with all confidence intervals including zero. The largest change occurred when the study by Harimochi S et al. was excluded, shifting the MD from -1.30 to -0.48, but the conclusion of no statistically significant difference remained unchanged.

**8. A univariable meta-regression for extubation time**

**8.1 control type (comparators)**


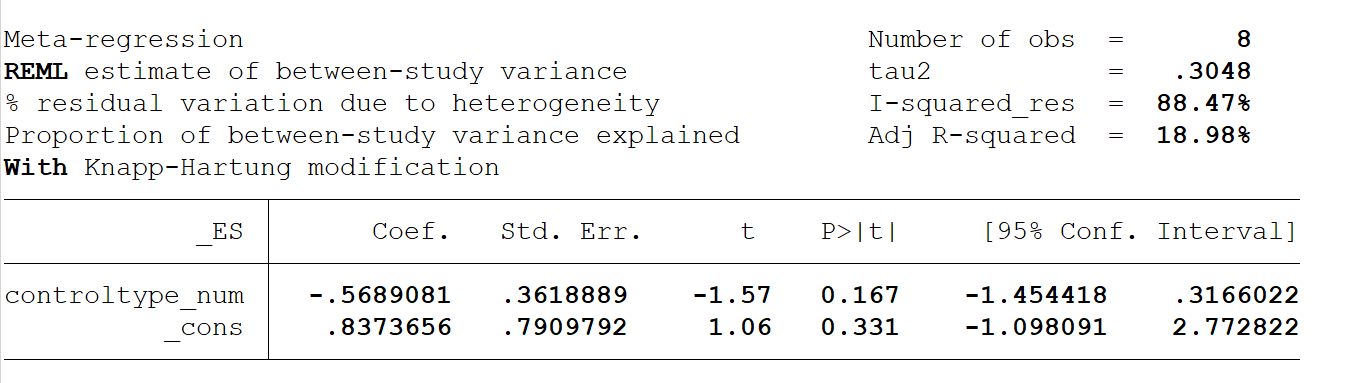


**8.2 country**


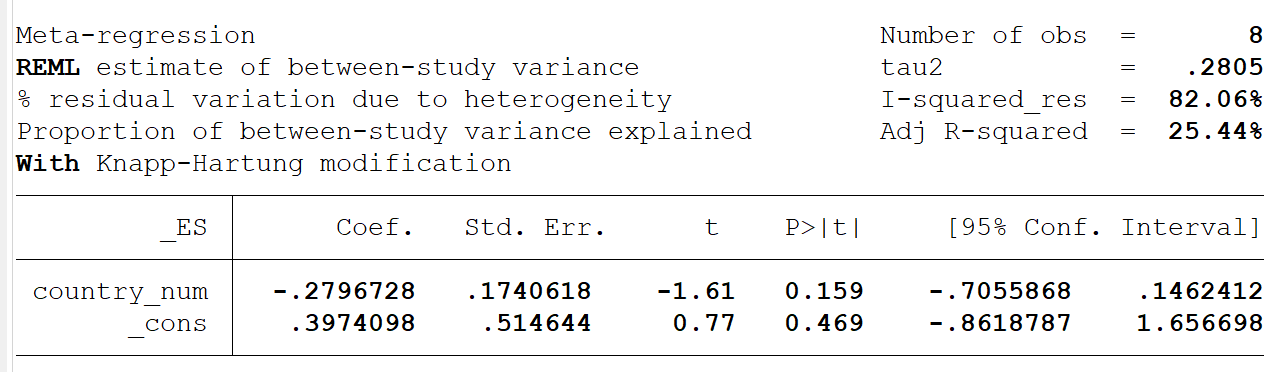


**8.3 study type (RCT or non RCT)**


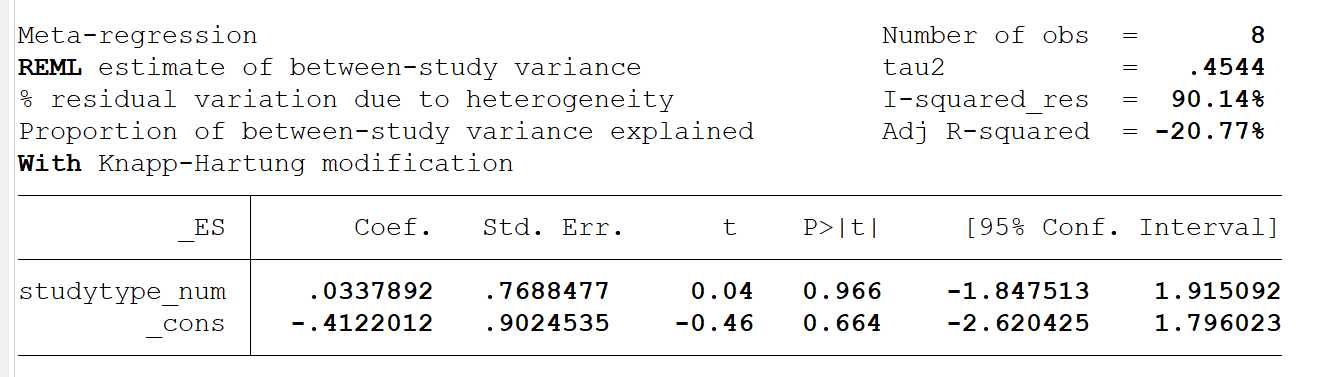


**8.4 age**


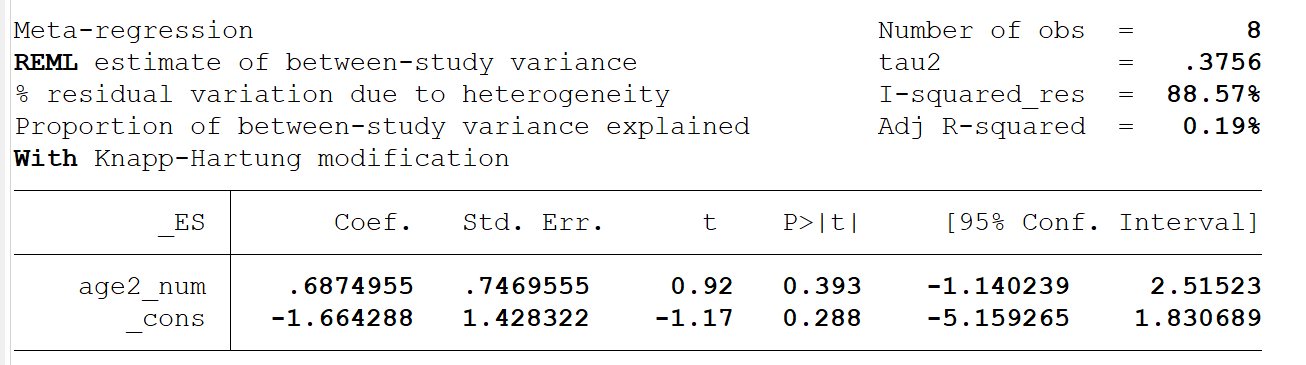


**8.5 surgery type**


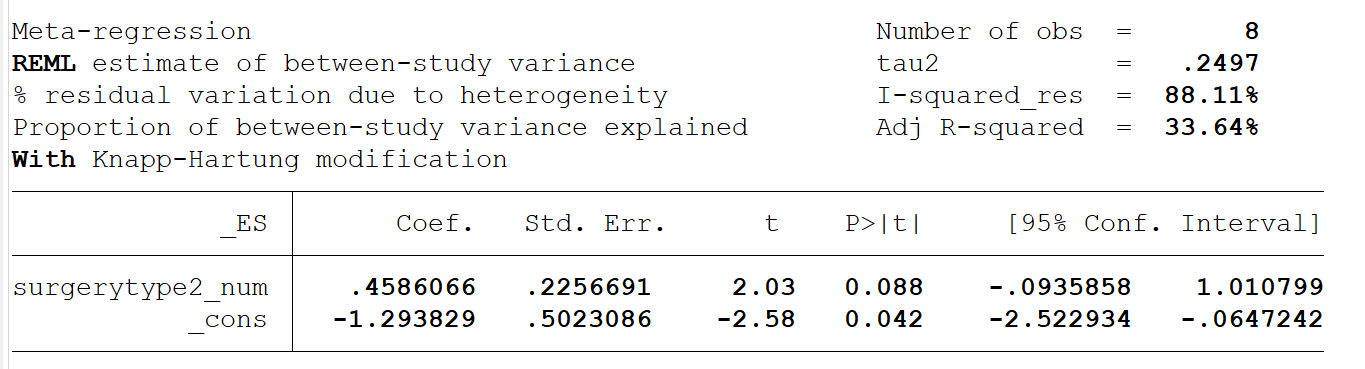


**8.6 the use of flumazenil**


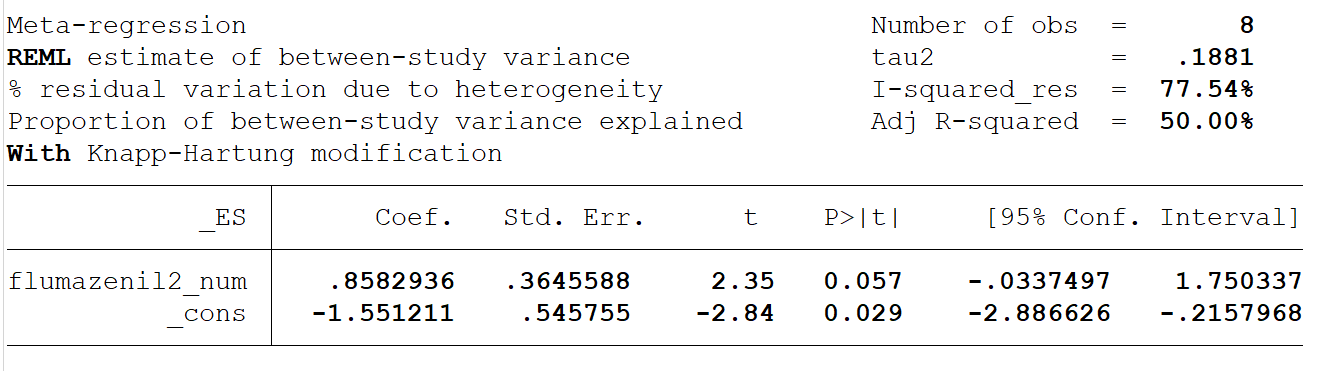


**9. To empirically assess the influence of study quality,** **a post-hoc sensitivity analysis excluding studies with a high overall risk of bias.**

**

**

The results of this analysis (pooled RR = 0.50, 95% CI: 0.19-1.31) were consistent with the primary analysis, suggesting that the overall finding of no significant difference in delirium risk was robust to the exclusion of higher risk studies. This provides some reassurance that the main conclusion is not solely driven by methodologically weaker studies."

**10. Subgroup analyses, with subgroups defined as RCT (25 studies) and non-RCT (5 studies), on the primary outcome delirium**


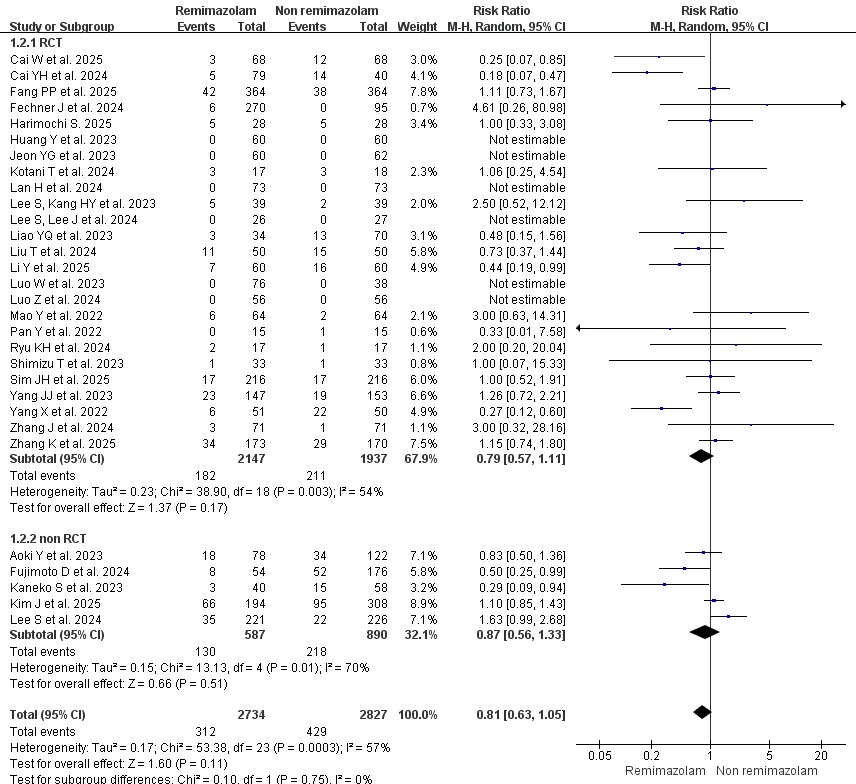


25 RCTs​ confirmed the robustness of our primary finding. The pooled risk ratio for the incidence of delirium was RR = 0.79 (95% CI: 0.57 to 1.11, P = 0.17), which is consistent with the main analysis that included all study designs (RR = 0.81, 95% CI: 0.63 to 1.05, P = 0.11). The point estimate and confidence interval remain virtually unchanged and non-significant.
