# Supplementary material for: The delirium dichotomy of remimazolam: a differential risk profile for emergence delirium versus postoperative delirium in surgical patients: a systematic review and meta-analysis
Source: Front Med (Lausanne). 2026 May 11;13:1841225. doi: 10.3389/fmed.2026.1841225 (PMC13199243; doi:10.3389/fmed.2026.1841225)
Supplement: Supplementary file 2 [file Table_1.docx]

The PRISMA 2020 reporting checklist

For checking that systematic review articles can be understood and used by everyone

| 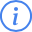 Note |
| --- |
| If you have not used a reporting guideline before, read about [how and why to use them](https:/resources.equator-network.org/about/reporting-guidelines.html) and check whether PRISMA 2020 is the [most applicable reporting guideline](https:/resources.equator-network.org/reporting-guidelines/prisma/index.html?#applicability) for your work.  Reporting guidelines are most useful when used early in research. When writing a manuscript or application, consider using the [Full Guidance](https:/resources.equator-network.org/reporting-guidelines/prisma/index.html) where you’ll see explanations and examples for each item.  After writing, demonstrate adherence by completing this checklist:   1. Specify where each item is described (see [Note 1](#sec-specify)). 2. Cite this checklist (See [Note 2](#sec-cite)). 3. Include your completed checklist as a supplement when submitting to a journal so that future readers can use it to find information. |

|  | Item Description | Location (or reason for not reporting) |
| --- | --- | --- |
| **Title and Abstract** |  |  |
| [1. Title](https:/resources.equator-network.org/reporting-guidelines/prisma/items/title.html?utm_source=prisma&utm_medium=checklist&utm_campaign=1_1) | Identify the report as a systematic review. | 1 |
| [2. Abstract](https:/resources.equator-network.org/reporting-guidelines/prisma/items/abstract.html?utm_source=prisma&utm_medium=checklist&utm_campaign=1_1) | Include all items from the *PRISMA 2020 for Abstracts* checklist. | 2 |
| **Introduction** |  |  |
| [3. Rationale](https:/resources.equator-network.org/reporting-guidelines/prisma/items/rationale.html?utm_source=prisma&utm_medium=checklist&utm_campaign=1_1) | Describe the rationale for the review in the context of existing knowledge. | 3 |
| [4. Objectives](https:/resources.equator-network.org/reporting-guidelines/prisma/items/objectives.html?utm_source=prisma&utm_medium=checklist&utm_campaign=1_1) | Provide an explicit statement of the objective(s) or question(s) the review addresses. | 3 |
| **Methods** |  |  |
| [5. Eligibility criteria](https:/resources.equator-network.org/reporting-guidelines/prisma/items/eligibility-criteria.html?utm_source=prisma&utm_medium=checklist&utm_campaign=1_1) | Specify the inclusion and exclusion criteria for the review and how studies were grouped for the syntheses. | 4 |
| [6. Information sources](https:/resources.equator-network.org/reporting-guidelines/prisma/items/information-sources.html?utm_source=prisma&utm_medium=checklist&utm_campaign=1_1) | Specify all databases, registers, websites, organisations, reference lists and other sources searched or consulted to identify studies. Specify the date when each source was last searched or consulted. | 4 |
| [7. Search](https:/resources.equator-network.org/reporting-guidelines/prisma/items/search.html?utm_source=prisma&utm_medium=checklist&utm_campaign=1_1) | Present the full search strategies for all databases, registers and websites, including any filters and limits used. | 4 and  Supplementary data |
| [8. Selection Process](https:/resources.equator-network.org/reporting-guidelines/prisma/items/selection-process.html?utm_source=prisma&utm_medium=checklist&utm_campaign=1_1) | Specify the methods used to decide whether a study met the inclusion criteria of the review, including how many reviewers screened each record and each report retrieved, whether they worked independently, and, if applicable, details of automation tools used in the process. | 4 |
| [9. Data collection process](https:/resources.equator-network.org/reporting-guidelines/prisma/items/data-collection-process.html?utm_source=prisma&utm_medium=checklist&utm_campaign=1_1) | Specify the methods used to collect data from reports, including how many reviewers collected data from each report, whether they worked independently, any processes for obtaining or confirming data from study investigators, and if applicable, details of automation tools used in the process. | 5 |
| 10. Data Items |  |  |
| [10a. Outcomes](https:/resources.equator-network.org/reporting-guidelines/prisma/items/data-items-outcomes.html?utm_source=prisma&utm_medium=checklist&utm_campaign=1_1) | List and define all outcomes for which data were sought. Specify whether all results that were compatible with each outcome domain in each study were sought (e.g. for all measures, time points, analyses), and if not, the methods used to decide which results to collect. | 5 |
| [10b. Other Variables](https:/resources.equator-network.org/reporting-guidelines/prisma/items/data-items-other-variables.html?utm_source=prisma&utm_medium=checklist&utm_campaign=1_1) | List and define all other variables for which data were sought (e.g. participant and intervention characteristics, funding sources). Describe any assumptions made about any missing or unclear information. | 5 |
| [11. Risk of bias in individual studies](https:/resources.equator-network.org/reporting-guidelines/prisma/items/risk-of-bias-in-individual-studies.html?utm_source=prisma&utm_medium=checklist&utm_campaign=1_1) | Specify the methods used to assess risk of bias in the included studies, including details of the tool(s) used, how many reviewers assessed each study and whether they worked independently, and if applicable, details of automation tools used in the process. | 5 |
| [12. Effect measures](https:/resources.equator-network.org/reporting-guidelines/prisma/items/effect-measures.html?utm_source=prisma&utm_medium=checklist&utm_campaign=1_1) | Specify for each outcome the effect measure(s) (e.g. risk ratio, mean difference) used in the synthesis or presentation of results. | 5-6 |
| 13. Synthesis Methods |  |  |
| [13a. Deciding which studies were eligible for each synthesis](https:/resources.equator-network.org/reporting-guidelines/prisma/items/synthesis-methods-eligibility.html?utm_source=prisma&utm_medium=checklist&utm_campaign=1_1) | Describe the processes used to decide which studies were eligible for each synthesis (such as tabulating the study intervention characteristics and comparing against the planned groups for each synthesis described in item 5. | 5-6 |
| [13b. Data preparation methods](https:/resources.equator-network.org/reporting-guidelines/prisma/items/synthesis-methods-data-preparation.html?utm_source=prisma&utm_medium=checklist&utm_campaign=1_1) | Describe any methods required to prepare the data for presentation or synthesis, such as handling of missing summary statistics, or data conversions. | 5-6 |
| [13c. Methods for tabulating or displaying results](https:/resources.equator-network.org/reporting-guidelines/prisma/items/synthesis-methods-tabulating-or-displaying-results.html?utm_source=prisma&utm_medium=checklist&utm_campaign=1_1) | Describe any methods used to tabulate or visually display results of individual studies and syntheses. | 5 |
| [13d. Synthesis methods](https:/resources.equator-network.org/reporting-guidelines/prisma/items/synthesis-methods-synthesis-methods.html?utm_source=prisma&utm_medium=checklist&utm_campaign=1_1) | Describe any methods used to synthesize results and provide a rationale for the choice(s). If meta-analysis was performed, describe the model(s), method(s) to identify the presence and extent of statistical heterogeneity, and software package(s) used. | 5-6 |
| [13e. Methods for exploring heterogeneity](https:/resources.equator-network.org/reporting-guidelines/prisma/items/synthesis-methods-exploring-heterogeneity.html?utm_source=prisma&utm_medium=checklist&utm_campaign=1_1) | Describe any methods used to explore possible causes of heterogeneity among study results (e.g. subgroup analysis, meta-regression). | 6 |
| [13f. Sensitivity analyses](https:/resources.equator-network.org/reporting-guidelines/prisma/items/synthesis-methods-sensitivity-analyses.html?utm_source=prisma&utm_medium=checklist&utm_campaign=1_1) | Describe any sensitivity analyses conducted to assess robustness of the synthesized results. | 6 |
| [14. Reporting bias assessment](https:/resources.equator-network.org/reporting-guidelines/prisma/items/reporting-bias-assessment.html?utm_source=prisma&utm_medium=checklist&utm_campaign=1_1) | Describe any methods used to assess risk of bias due to missing results in a synthesis (arising from reporting biases). | 6 |
| [15. Certainty assessment](https:/resources.equator-network.org/reporting-guidelines/prisma/items/certainty-assessment.html?utm_source=prisma&utm_medium=checklist&utm_campaign=1_1) | Describe any methods used to assess certainty (or confidence) in the body of evidence for an outcome. | 5-6 |
| **Results** |  |  |
| 16. Study Selection |  |  |
| [16a. Results of the search and selection process](https:/resources.equator-network.org/reporting-guidelines/prisma/items/study-selection-search-results.html?utm_source=prisma&utm_medium=checklist&utm_campaign=1_1) | Describe the results of the search and selection process, from the number of records identified in the search to the number of studies included in the review, ideally using a flow diagram. | 6 |
| [16b. Excluded studies](https:/resources.equator-network.org/reporting-guidelines/prisma/items/study-selection-excluded-studies.html?utm_source=prisma&utm_medium=checklist&utm_campaign=1_1) | Cite studies that might appear to meet the inclusion criteria, but which were excluded, and explain why they were excluded. | 6 |
| [17. Study characteristics](https:/resources.equator-network.org/reporting-guidelines/prisma/items/study-characteristics.html?utm_source=prisma&utm_medium=checklist&utm_campaign=1_1) | Cite each included study and present its characteristics. | 6-7 |
| [18. Risk of bias in studies](https:/resources.equator-network.org/reporting-guidelines/prisma/items/risk-of-bias-in-studies.html?utm_source=prisma&utm_medium=checklist&utm_campaign=1_1) | Present assessments of risk of bias for each included study. | 7 |
| [19. Results of individual studies](https:/resources.equator-network.org/reporting-guidelines/prisma/items/results-of-individual-studies.html?utm_source=prisma&utm_medium=checklist&utm_campaign=1_1) | For all outcomes, present, for each study: (a) summary statistics for each group (where appropriate) and (b) an effect estimate and its precision (e.g. confidence/credible interval), ideally using structured tables or plots. | 6-7 |
| 20. Results of Synthesis |  |  |
| [20a. Summary of studies](https:/resources.equator-network.org/reporting-guidelines/prisma/items/results-of-syntheses-summary-of-studies.html?utm_source=prisma&utm_medium=checklist&utm_campaign=1_1) | For each synthesis, briefly summarise the characteristics and risk of bias among contributing studies. | 6-7 |
| [20b. Statistical results](https:/resources.equator-network.org/reporting-guidelines/prisma/items/results-of-syntheses-statistical-results.html?utm_source=prisma&utm_medium=checklist&utm_campaign=1_1) | Present results of all statistical syntheses conducted. If meta-analysis was done, present for each the summary estimate and its precision (e.g. confidence/credible interval) and measures of statistical heterogeneity. If comparing groups, describe the direction of the effect. | 7-11 |
| [20c. Heterogeneity](https:/resources.equator-network.org/reporting-guidelines/prisma/items/results-of-syntheses-heterogeneity.html?utm_source=prisma&utm_medium=checklist&utm_campaign=1_1) | Present results of all investigations of possible causes of heterogeneity among study results. | 8 |
| [20d. Sensitivity analyses](https:/resources.equator-network.org/reporting-guidelines/prisma/items/results-of-syntheses-sensitivity-analyses.html?utm_source=prisma&utm_medium=checklist&utm_campaign=1_1) | Present results of all sensitivity analyses conducted to assess the robustness of the synthesized results. | 8 |
| [21. Risk of reporting biases in syntheses](https:/resources.equator-network.org/reporting-guidelines/prisma/items/risk-of-reporting-biases-in-syntheses.html?utm_source=prisma&utm_medium=checklist&utm_campaign=1_1) | Present assessments of risk of bias due to missing results (arising from reporting biases) for each synthesis assessed. | No |
| [22. Certainty of evidence](https:/resources.equator-network.org/reporting-guidelines/prisma/items/certainty-of-evidence.html?utm_source=prisma&utm_medium=checklist&utm_campaign=1_1) | Present assessments of certainty (or confidence) in the body of evidence for each outcome assessed. | 7-11 |
| **Discussion** |  |  |
| 23. Discussion |  |  |
| [23a. General interpretation of the results](https:/resources.equator-network.org/reporting-guidelines/prisma/items/discussion-general-interpretation.html?utm_source=prisma&utm_medium=checklist&utm_campaign=1_1) | Provide a general interpretation of the results in the context of other evidence. | 11-12 |
| [23b. Limitations of included evidence](https:/resources.equator-network.org/reporting-guidelines/prisma/items/discussion-limitations-of-included-evidence.html?utm_source=prisma&utm_medium=checklist&utm_campaign=1_1) | Discuss any limitations of the evidence included in the review. | 16 |
| [23c. Limitations of the review processes](https:/resources.equator-network.org/reporting-guidelines/prisma/items/discussion-limitations-of-review-process.html?utm_source=prisma&utm_medium=checklist&utm_campaign=1_1) | Discuss any limitations of the review processes used. | 16 |
| [23d. Implications](https:/resources.equator-network.org/reporting-guidelines/prisma/items/discussion-implications.html?utm_source=prisma&utm_medium=checklist&utm_campaign=1_1) | Discuss implications of the results for practice, policy, and future research. | 12-14 |
| **Other Information** |  |  |
| 24. Registration and Protocol |  |  |
| [24a. Registration](https:/resources.equator-network.org/reporting-guidelines/prisma/items/registration-and-protocol-registration.html?utm_source=prisma&utm_medium=checklist&utm_campaign=1_1) | Provide registration information for the review, including register name and registration number, or state that the review was not registered. | 4  Registered at PROSPERO on September 19, 2025 (CRD420251138775). |
| [24b. Protocol](https:/resources.equator-network.org/reporting-guidelines/prisma/items/registration-and-protocol-protocol.html?utm_source=prisma&utm_medium=checklist&utm_campaign=1_1) | Indicate where the review protocol can be accessed, or state that a protocol was not prepared. | PROSPERO website |
| [24c. Amendments](https:/resources.equator-network.org/reporting-guidelines/prisma/items/registration-and-protocol-amendments.html?utm_source=prisma&utm_medium=checklist&utm_campaign=1_1) | Describe and explain any amendments to information provided at registration or in the protocol. | No |
| [25. Support](https:/resources.equator-network.org/reporting-guidelines/prisma/items/support.html?utm_source=prisma&utm_medium=checklist&utm_campaign=1_1) | Describe sources of financial or non-financial support for the review, and the role of the funders or sponsors in the review. | 18 |
| [26. Competing Interests](https:/resources.equator-network.org/reporting-guidelines/prisma/items/competing-interests.html?utm_source=prisma&utm_medium=checklist&utm_campaign=1_1) | Declare any competing interests of review authors. | 18 |
| [27. Availability of data, code, and other materials](https:/resources.equator-network.org/reporting-guidelines/prisma/items/availability-of-materials.html?utm_source=prisma&utm_medium=checklist&utm_campaign=1_1) | Report which of the following are publicly available and where they can be found: template data collection forms; data extracted from included studies; data used for all analyses; analytic code; any other materials used in the review. | 19 |

## 1 How to specify where content is

Tell the reader where they can find information. E.g.,

- Results; paragraph 2
- Methods, Participants; paragraphs 1 & 2.
- Table 3
- Supplement B, para. 4

If you have chosen not to describe an item, explain why. You can do this in the checklist, or as a note below it.

You can describe items in the article body, or in tables, figures, or supplementary materials, and should prioritize items you feel are most important to your intended audience. The order of items in your manuscript does not need to match the order of items in this checklist. You can decide how best to structure your work.

## 2 How to cite

Describe how you used PRISMA 2020 at the end of your Methods section, referencing the resources you used e.g.,

‘We used the PRISMA 2020 reporting guideline(1) to draft this manuscript, and the PRISMA 2020 reporting checklist(2) when editing, included in supplement A’

If you use a reporting checklist, remember to include it as a supplement when publishing so that readers can easily find information and see how you have interpreted the guidance.

1. Page MJ, McKenzie JE, Bossuyt PM, Boutron I, Hoffmann TC, Mulrow CD, et al. The PRISMA 2020 statement: An updated guideline for reporting systematic reviews. PLOS Medicine [Internet]. 2021 Mar;18(3):e1003583. Available from: <https://journals.plos.org/plosmedicine/article?id=10.1371/journal.pmed.1003583>

2. Page MJ, McKenzie JE, Bossuyt PM, Boutron I, Hoffmann TC, Mulrow CD, et al. The PRISMA 2020 reporting checklist. In: Harwood J, Albury C, Beyer J de, Schlüssel M, Collins G, editors. The EQUATOR network reporting guideline platform [Internet]. The UK EQUATOR Centre; 2025. Available from: [https:/resources.equator-network.org/reporting-guidelines/prisma/prisma-checklist.docx](https://https:/resources.equator-network.org/reporting-guidelines/prisma/prisma-checklist.docx)
